# Experience of adolescents/young adults with chronic kidney disease and of their families during transition of care

**DOI:** 10.1590/1980-220X-REEUSP-2024-0365en

**Published:** 2025-06-20

**Authors:** Júlia Rudzinski Roveri, Maiara Rodrigues dos Santos, Ana Márcia Chiaradia Mendes-Castillo, Aline Oliveira Silveira, Regina Szylit, Maira Deguer Misko

**Affiliations:** 1 Universidade Estadual de Campinas, Faculdade de Enfermagem, Campinas, SP, Brazil.; 2 Universidade de São Paulo, Escola de Enfermagem, Departamento de Enfermagem Materno-Infantil e Psiquiátrica, São Paulo, SP, Brazil.; 3 Universidade de Brasília, Faculdade de Ciências da Saúde, Departamento de Enfermagem, Brasília, DF, Brazil.

**Keywords:** Renal Insufficiency, Chronic, Chronic Disease, Transition to Adult Care, Adolescent, Young Adult, Pediatric Nursing

## Abstract

**Objective:**

To understand the experience of adolescents/young adults with chronic kidney disease and of their families during transition of care.

**Method:**

Qualitative study, based on the assumptions of the Grounded Theory and of the theoretical framework of Symbolic Interactionism. Interviews were held with eight families of adolescents/young adults diagnosed with chronic kidney disease, in the process of transition of care, followed in a public teaching hospital, during the years 2019 and 2020. Data were obtained through participant observation, semi-structured interviews, and consultations of clinical records.

**Results:**

Comparative analysis of the data allowed us to reveal the family’s experience in the process, represented by the categories “having to move on to the next phase”, in which participants describe the need to strengthen the independence of the adolescent/young adult through the change of roles within the family and “exploring the new world”, where the autonomy of the teenager/young adult must be encouraged.

**Conclusion:**

The study highlighted the importance of individualized transition plans, collaboration between family and health team, and enhancement of adolescents and young adults’ independence and self-care.

## INTRODUCTION

The prevalence of chronic diseases in adolescence has increased considerably in recent decades. It is estimated that approximately 10% of adolescents in Western countries live with chronic health conditions nowadays^(1)^. Continuous technological progress in the medical field has contributed to better prognoses, allowing these young people to reach adulthood, increasing the need to transition from pediatric care to the adult-oriented health system^(2,3)^.

The most widely used concept in the literature for the term “care transition” is that of the Society for Adolescent Medicine, which defines it as “an intentional and planned movement of adolescents and young adults with chronic conditions from a pediatric service to adult-oriented health services”^(4,5)^. The adolescent/young adult living with a chronic disease, besides the expected changes for their age, will also have to face changes in their health care and in the way it will be provided, adding complexity to an already turbulent transition period^(6,7)^.

Although several recommendations have been made in recent years, the transition to adult care still represents a challenge for adolescents, healthcare workers, and family members. This process requires a change in family dynamics, in which new roles and responsibilities are assumed among its members^(8)^. Many adolescents experience the transition in a disjointed way, facing difficulties both in leaving the familiar pediatric environment and in adapting to the adult environment. Pediatric professionals are reluctant to transfer responsibility to patients, while the adult healthcare staff is not always prepared to deal with young people^(1)^.

Transition to adult care should consider the complexity of the illness, the family context, and the adolescent’s readiness to take responsibility for his or her health, rather than relying solely on chronological age^(9)^. An abrupt transition, with no planning or coordination, can lead to helplessness, compromise adherence to treatment, and worsen the clinical condition. Therefore, it is essential that the healthcare team develops a gradual and structured plan to ensure continuity of care, promoting better adherence to treatment and preventing future complications of the chronic disease^(6,7)^.

For this to be possible, it is understood that it is extremely important to know the feelings and expectations of the adolescent and their family who will soon be moving into adult care, as well as the experience lived by the young adult who is already being followed up in the adult outpatient clinic and their family. We hope that this knowledge allows for the healthcare professional to outline an individualized and organized care transition program , taking into account the family dynamics, the illness, and the sociocultural context in which the adolescent is inserted^(10,11)^.

Thus, this study aimed to know the family and/or the adolescent/young adult’s experience of chronic kidney disease during their care transition process from pediatrics to an adult service.

## METHOD

### Design of Study

Qualitative study, supported by the assumptions of the methodological framework of Grounded Theory, developed from the perspective of Symbolic Interactionism as a theoretical framework. This methodology was chosen for its ability to guide the collection, organization and analysis of data, with the aim of developing a theory, hypothesis, or concept based on the information obtained during the research. It seeks to extract meaning from lived experiences, with an emphasis on social interactions. Furthermore, it allows the construction of interrelated categories that contribute to the formation of a theoretical structure, expanding the understanding of the topics studied, exploring new perspectives and clarifying the experiences analyzed^(12)^. Recommendations from the Consolidated Criteria for Reporting Qualitative Research (COREQ) guide^(13)^ directed the construction of this work.

### Local

Data collection was carried out at the Pediatric Nephrology Outpatient Clinic and the Adult Nephrology Outpatient Clinic, both belonging to the Integrated Nephrology Center (CIN), unit that integrates a tertiary-level public hospital complex, focused on teaching and research activities, located in an inland city of the state of São Paulo. This unit is exclusively for the care of patients with chronic kidney disease, with 30% of pediatric patients and 70% of adults. Outpatient monitoring covers all therapeutic modalities and provides care for all stages of kidney disease, from non-surgical treatment to kidney transplant. The medical team determines which adolescents should care transition to adult services, using as main criteria being 18 years old and having clinical stability. Patients with unstable health conditions and families with significant social or emotional vulnerability may have their transition of care delayed until clinical or family stability is achieved, which may occur after the patient is 20 years old.

### Population

Participants in this research included adolescents (ages 12–18) and young adults (ages 19–24) with chronic kidney disease, as well as their family members. The selection of this age range aimed to encompass the complete experience of the care transition, considering all stages of adolescence equally. Adolescents who are still receiving pediatric care can share their expectations for the future, while young adults, already receiving care in an adult outpatient clinic, express their feelings about the transition already experienced. It should be noted that all adolescent and young adult participants underwent conservative treatment in the outpatient clinic, with the exception of members of families 4 and 5, who had already undergone kidney transplantation.

Currently, the definition of family varies according to each individual’s reference, with their values and beliefs being considered. In other words, family is defined by its own members, regardless of blood ties. However, the family system is not only made up of people who have emotional bonds, but also of individuals who perform a cultural and social function within this system^(14)^, and this was the definition used in the construction of this work.

The following inclusion criteria were used: being a family member of an adolescent or young adult with chronic kidney disease who has experienced or will experience the care transition process at the CIN; being an adolescent or young adult diagnosed with chronic kidney disease who has experienced or will experience the care transition process at the CIN. The exclusion criteria were: family members, adolescents and young adults with neurological or speech deficits, making it impossible to carry out interviews; adolescents or young adults who were hospitalized throughout the data collection phase.

Therefore, 8 families participated in the research, with a total of 14 family members interviewed: six adolescents and one young adult with chronic kidney disease, four mothers, two fathers and a sister. It should be noted that the young adult from family 05 did not participate in the research due to delayed neuropsychomotor development. In the case of family 06, only the young adult participated in the research because he no longer attends the service with a companion. None of the families invited to participate in the study refused the invitation. Participants are described in Chart 1.

**Chart 1:** Researchers’ participants characterization – Campinas, SP, Brazil, 2020.

Sample Group	Family	Name	Age	Kinship	Diagnosis	Transition time
1 – Pediatric Outpatient Clinic	Family 1	Rosana	42	Mother	Nephrotic Syndrome	No estimation
		Marcela	17	Patient		
	Family 2	Clara	45	Mother	Nephrotic Syndrome	Last pediatric consultation
		Eduardo	18	Patient		
	Family 3	André	43	Father	Glomerulopathy	Last pediatric consultation
		Laís	19	Patient		
	Family 4	Mara	48	Mother	Nephropathic Cystinosis	No estimation
		Vitor	20	Patient		
2 – Adult Outpatient Clinic	Family 5	Francisco	40	Father	Chronic Renal Insufficiency	1 month
	Family 6	Antônio	18	Patient	Nephrotic Syndrome	5 months
	Family 7	Paula	46	Mother	Chronic Renal Insufficiency	5 months
		Valeria	18	Patient		
	Family 8	Milena	25	Sister	Chronic Renal Insufficiency	1st consultation
		Caio	18	Patient		

### Sample Definition

Data collection was completed upon reaching theoretical saturation, stage in which recurrence and lack of new elements indicate that the deepening of the theory was sufficient. This concept is widely used in qualitative research in the health field. Thus, the number of participants was not pre-defined, being determined as the data was collected and analyzed^(15)^.

The sample group called “Pediatric Outpatient Clinic” consisted of four families who were being monitored at the pediatric nephrology outpatient clinic, but who had already been informed by the health team that the transition process to adult care was close to being completed. This first moment aimed to learn how the teenager/young adult and his/her families experienced the transition of care while it was still a possibility, what their feelings and expectations were regarding this. The second sample group consisted of four families who had already experienced the transition process to adult care and were starting or were already receiving follow-up at the adult outpatient clinic, which was essential to broaden the perspective of the family’s experience in the transition process. It is important to emphasize that the sample groups are distinct and do not overlap; that is, the families in the first sample group did not participate in the research at the second time and vice versa.

### Data Collection

Data collection was carried out from August 2019 to March 2020, using participant observation methods, semi-structured interviews, and consultation of medical records.

Participant observation is a strategy that allows the researcher to immerse themselves in the situation under investigation, following verbal and non-verbal expressions, in addition to contributing to the identification of details that, until then, had not been noticed or reported during the interviews. Observation was carried out both in the waiting room and during nursing consultations preceding medical consultations. Each family was monitored, on average, for 60 minutes. These observations were recorded in a field diary at the time they were made, being called “observation notes”.

The semi-structured interview was conducted by the researcher in charge and consisted of two sections: the first aimed at obtaining sociodemographic information and characterizing the family and the second composed of the guiding question “How has it been for you to experience the transition process of care from a pediatric service to an adult service with your child/family member?” The speeches arising from this issue were permeated by new questions, such as, “How did this situation make you feel?” and “What do you believe could have helped you in this situation/moment?”, to deepen the description of the experience lived. The interviews lasted an average of 30 to 40 minutes and took place only once with each participant.

Contact with participants was made in the waiting area of the pediatric or adult outpatient clinic, while they waited for medical or nursing care. The researcher was introduced to the family by professionals at the outpatient clinic, without establishing any prior connection or contact with these families, either before or after the interview. Only the researcher collected the data, and she did not have any connection with the study site, which helped to minimize possible discomfort or biases in accessing research subjects.

The interview with each family was carried out individually, respecting the privacy of the interviewee, in the place and at the time of the family’s preference, and all interviewees chose to carry out the interview in the clinic where they were treated, in a private room previously reserved by the researcher for this purpose. The family was given the option of having the interviews conducted without the presence of the adolescent/young adult and the reverse was also done; however, no family used this resource.

The teenagers were invited to participate in the interview, and could choose to attend accompanied by their family members or individually. Everyone decided to participate together with their families and expressed themselves openly, sharing their feelings and expectations. Participants were given pseudonyms to ensure confidentiality of their identities.

The main researcher conducted data collection in a comprehensive manner and, to this end, underwent training to conduct interviews with families, provided by the research group to which she belongs. The interviews were recorded on a digital device and then transcribed in full.

The transcripts were reviewed by the research group members and the participants did not have access to the transcripts.

Consultations were also carried out on patients’ physical and electronic medical records, with the aim of obtaining information such as diagnosis, length of follow-up in the service, treatment, and prognosis of the disease.

### Data Analysis and Treatment

The recorded statements were transcribed in full manually and analyzed using the continuous comparative method, through which the categories were compared to identify similar or different concepts, simultaneously with data collection. The categories have been revised by the team members.

Data analysis was conducted based on the principles of Grounded Theory, using open, axial, and selective coding. Thus, the first stage of the analytical process was open coding, which consists of the researcher carefully reading each sentence said by the interviewee, followed by reflection and conceptualization of the sentence. At this stage, expressions were assigned to the interview fragments, with the aim of forming codes. This process involved sentence-by-sentence and word-by-word analysis of the interview, considering the research questions and objectives^(16)^. As the questions were answered, it was possible to identify the first properties, which enabled the formation of the categories presented in this work.

Axial coding was the second phase of the analysis, characterized by the process of linking categories to their subcategories, with the aim of generating more detailed and complete explanations about the phenomena. Because connections between categories can be quite implicit, Strauss and Corbin^(16)^ suggest the development of a scheme that can be used to structure emerging relationships. In this study, the type called “process” was adopted for axial coding. Strauss and Corbin^(16)^ define it as a series of evolutionary sequences of action/interaction that occur over time and space, changing or remaining constant at certain moments, in response to the situation or context. Thus, the process in the data is represented by events and occurrences that may or may not unfold in continuous sequences.

Selective coding is the third phase of analysis, in which we sought to integrate and improve the theory. As in the previous stages of analysis, this integration involves an interaction between the analyst and the data, as it encompasses the evolution of thinking over time, through immersion in the data and the set of results obtained.

The data were then compared in search of similarities and differences. This process allowed us to analyze in detail how words and phrases were used, associating them with feelings and experiences, thus helping in the interpretation of data, forming groupings of more abstract concepts, which are called categories and are exemplified in Chart 2.

**Chart 2:** Categorization Example – Campinas, SP, Brazil, 2020.

Codes	Category
– Staying a little expectant. – Having some anxiety. – Knowing that they are not the same professionals, but believing that they will have the same encouragement. – Believing that it will add more. – Receiving the news that I was going to the adult outpatient clinic. – Looking forward to the transition.	HAVING TO ADVANCE TO THE NEXT STAGE

### Ethical Aspects

The project was submitted to the Research Ethics Committee of the Universidade Estadual de Campinas, being approved under opinion number 3.276.133. It should be highlighted that the requirements of Resolution 466/2012, of the National Health Council, were strictly followed. All participants were informed about the objectives, procedures and methods of the study, especially regarding the use of the recorder during the interviews, the guarantee of confidentiality of information, the right to anonymity, and the freedom to choose whether or not to participate in the research. Teenagers and their families were invited to integrate the study and, after reading the Free and Informed Assent Form (for participants under 18 years of age) and Free and Informed Consent Form (for those over 18 years old and all family members), doubts were clarified and the signature of the terms by those involved was requested.

## RESULTS

The meanings given by family members to the experience of going through the transition process to adult care were understood after continuous evaluation and comparison of data. Therefore, the process reflecting family experience consists of two categories: “Having to advance to the next stage” and “Exploring the new world”.

### Having to Advance to The Next Stage

The experience of families of adolescents and/or young adults with chronic kidney disease during the care transition process is closely linked to the family adaptation process, particularly when the diagnosis is made in childhood. This adaptation process involves a combination of emotional and practical factors, reflecting both changes in the family’s daily life and the management of emotions in the face of a chronic and potentially debilitating condition.

Many families reported a decrease in emotional fragility when they began follow-up in the pediatric service. This support from the healthcare team contributed to coping with the disease and establishing a treatment routine. Living together in the outpatient clinic strengthened the bonds of affection between family members and health workers, reducing the emotional burden that the disease carries.

*“There’s the personal side here too. Because I’ve been coming here for 16 years, so they’ve known me since I was little.” [Eduardo, 18 years old.]*

*“All the nurses are friends with him, they all know him since he was a child. ‘Oh Joãozinho, you’re already big, you’re going to become an adult now!’. It now looks like this: ‘Wow, are you going to visit the adult clinic? No, it can’t be!’” [Milena, sister]*

*“Marcela waits in the waiting room with her mother, while looking at her cell phone. She is called by the nurse and enters the nursing office alone. Inside the office, Marcela already knows the procedure: she takes off her shoes and her blouse tied around her waist. She steps on the scale and the nurse, while writing down her weight, asks about her boyfriend, with whom Marcela recently started a relationship. Marcela starts to talk about her last weekend and the conversation flows like two friends.”[field notes taken by the researcher based on participant observation]*

During care transition, the family will break emotional ties. Upon leaving pediatrics, the family will be leaving behind years of built social interactions, shared experiences and learning, sad and happy moments that made up their trajectories, to enter a world with new meanings. Families report insecurity due to the lack of support and information, as well as fear and sadness in moving on to the next phase, especially when they do not have the necessary instructions and rules to face this change.

*“I don’t want to go* (to the adult outpatient clinic)*. I feel sad, I don’t want to go. I don’t even think about it much, I don’t want to go there, I like it here.” [Marcela, 17 years old.]*

*“Just fear. I don’t know what it’s like there, I don’t know what it’s going to be like. Just fear.” [Laís, 19 years old]*

*“The medical board on the children’s side is very good, there are some doctors there, wow, very good. If I could, I would leave him here (in the children’s clinic). You see that it is more affectionate, like this. I didn’t like them very much, the adults there, who didn’t pay much attention.” [Francisco, father]*

The possibility of changes in treatment and health routines brings to the family the feeling that the care transition process may entail a risk to the continuity of treatment, or add new restrictions to daily life, as is the case with hemodialysis.

*“He talks a lot about this: ‘Will there be much difference?’ (...) Will I change any medication? Will I change any routine?’ He doesn’t know yet.” [Milena, sister.] “I think my biggest fear is getting there and, you know, hemodialysis. Because here, I don’t know how it is there, but here they give medicine, because caring for a child is different than caring for an adult. They try to give the child privacy to continue growing, but not there.” [Laís, 19 years old.]*

Not only are the procedures and routines different in the adult outpatient clinic, but also the physical space and personal interactions in the new environment, which has a significant impact on the family. This is because relationships tend to be more distant and consultations more formal. Many families report feeling unprepared to face this change in team and outpatient clinic, while at the same time feeling pressured to move on to the next phase and accept the transition.

The young adult and his family will have to interact with a new and unknown health team, with whom they will have to establish relationships of trust similar to those experienced in the pediatric environment. However, in families, there is a fear that these new relationships will become more difficult and rigid. Furthermore, the natural difficulty in interacting typical of adolescence, enhanced by the presence of a chronic illness, is a concern frequently expressed by families experiencing this process.

*“You already know (pediatrics), they already know who you are, they already know your way. One minute you are happy, the next minute you are discouraged. So it seems like you already become friends there. I’ve been to the other side (adult outpatient clinic) several times, but it’s all a bit rude.” [Mara, mother]*

*“I think the doctor (at the adult clinic) won’t treat you the same. Maybe he’s a little stricter, don’t ask about personal life.” [Eduardo, 18 years old.]*

*“At first I found it a little strange, I thought it was a little different. Here (pediatrics) they did it one way, there it’s another. The way of weighing is different.” Antônio, 18 years old.*

This process generates anxiety and suffering in the families, who now realize that they will have to develop new skills so that the teenager or young adult gain a voice and be the narrator of his/her story. The fear of not establishing ties with the new team is deeply related to the fear of introducing oneself to a previously unknown group.

*“Ah, it’s just that my mother usually speaks for me. Now I’m trying to talk to myself, now. That’s what’s kind of weird.” [Valeria, 18 years old.]*

Families fear not being able to replicate the procedures and treatments carried out during follow-up in pediatric nephrology for the adult team, which could compromise the follow-up and health of the adolescent. The continuity of treatment seems to be at risk, and they feel they will have to face this journey alone and with little information.

*“As for the transition (…) I’m not a doctor to say, to tell my story like that, about what happened, right? I know it happened, but I won’t be able to talk about the technical part. Say: ‘he started with nephrotic syndrome, all this happened, he has a case like that’, tell about my case, you know? And have it ready for the doctor to pick it up and say: ‘ah, this one is Eduardo!’. Already know who Eduardo is. It would make me feel a little more at ease, because he would already know a little about my story. Of course he wouldn’t know everything, because follow-up is just starting.” [Eduardo, 18 years old.]*

In the “Having to Move on to the Next Stage” category, the family faces the inevitable transition of care, seeking to build a new identity while interacting with unfamiliar people and situations. Throughout this process, the family notices the need to support the adolescent’s independence, seeking to establish our symbols based on the new relationships that begin to be established, thus ensuring the continuity of health care. That said, there is no turning back and adolescents and/or young adults and their families are now experiencing a new moment, starting with the transition of care, represented by the second category, named “Exploring the new world”.

### Exploring the New World

The transition to adult care represents the beginning of a new era in the family’s life and is one of the biggest obstacles since diagnosis. The “Exploring the New World” category represents the family’s effort to gather the essential tools to adapt to a different context, characterized by new structures, rules and routines. This period is also notable for the reorganization of family roles, which must adapt to the demands of this new reality.

With the child’s diagnosis, the family reorganizes itself so that one of its members, usually the mother, assumes central care and dedicates herself almost exclusively to the child, supervising treatment and health monitoring. In this process, these mothers begin to play a new role in the family dynamics: that of caregiver for a child with a chronic illness. This configuration usually lasts throughout childhood, but, in the transition to adulthood, many mothers return to their professional activities and need to gradually transfer control and responsibilities for treatment to their children, now teenagers, challenging them to take responsibility for their own clinical condition.

*“It’s been two months now that I’ve returned to my normal routine, because until then...before he got sick, I was working, then I wasn’t...I did something like here and there, to fill the time (...) but now I’m really working.” [Clara, mother]*

*“(The mother) controls everything, leaves the medicines cut to the right milligram, she sticks to the schedule (...) I don’t know if it’s the teenage phase, but he’s very reluctant. So, you have to push him.” [Milena, sister.]*

As teenagers enter adulthood, they gain responsibilities and autonomy, although these are not always accompanied by the emotional maturity or readiness necessary to take control of the situation. Care transitioning requires the patient to take responsibility for their own illness and treatment, as well as for the consequences resulting from non-adherence to treatment. At this stage, the family often questions the adolescent’s readiness to take on a more active role in their care, while at the same time fearing possible worsening of the clinical condition. Many family members feel insecure and even threatened by the possibility of losing control over the adolescent’s health, which can generate concerns about the continuity of treatment and the risk of worsening of the clinical condition.

*“It’s weird that I let her come alone. Because the day she said: ‘Mom, I’m going to the adult and you can’t go!’ But wait, how long did I follow her? I took care of her, gave medicine, and such. ‘But mom, I’m 18, it’s time to cut the cord, right?’ [Paula, mother].*

*“Today he will sit in a room with a doctor, and the first time I will go with him, but next time I may not go, so I will have to believe that everything the doctor tells him, he will have to pass on to me or my mother.” [Milena, sister].*

*“Usually my mother speaks for me. Now I’m trying to talk for myself. That’s kind of weird.” [Valeria, 18 years old.]*

The adult clinic is often described by family members as “real life,” while pediatrics is presented as a “make-believe” world. These family members carry with them the anguish of seeing their young people grow up and enter the real world and have to take control of their illness.

*“This is a new phase of life for her, which I think is more difficult. For her to get adapted, her adaptation there. Because she’s going to have to deal with people, that whole child, pre-teen thing is over. Things are more serious for adults, in all aspects, not just here.” [Rosana, mother.]*

In this change of roles, family members leave the main role of care and take on the role of support, while the adolescent turns to himself in search of tools and resources to develop autonomy and face the fear of not feeling prepared for this moment. In an adult environment, family members will be seen as support, but it will be the young adult who will have to answer for their decisions and behaviors when faced with the disease. They mention the fear and anxiety about the charges they imagine they will receive when the transition process from pediatric to adult care actually takes place.

*“Here (pediatrics) I was kind of: ‘Okay, you won’t have the job yet. The weight doesn’t go to you, it goes to your companion’. But now the weight is on me. Anything happening, it will be my fault.” [Caio, 18 years old.]*

*“From the moment she turns 18, a lot of things will change in her life. She will have to face things head on, she is no longer a child, she no longer has a certain privilege here. The thing is more serious.” [Rosana, mother.]*

*“Now the responsibility weighs on him. When we follow, when we are close it is easier, because they come to us, we demand and push them. Now, when it’s up to him, for him to take it (...) I feel for him, because now he sees that the responsibility is his. It’s not every time that we’ll be able to go in, that we’ll be able to follow, that now the responsibility is his. So in some sense it will be good for maturation.” [Milena, sister.]*

This analytical process explores, in the family’s experience, the dynamics between facing the inevitable transition to the adult outpatient care clinic and dealing with the rupture of the ties established in the pediatric service. This rupture is accompanied by fear of possible consequences, such as discontinuation of treatment and worsening of the adolescent’s clinical condition. In this context, the adolescent or young adult emerges as the protagonist of their treatment, seeking greater autonomy and independence.

## Discussion

The results obtained in this study expand the understanding of how adolescents and young adults with chronic kidney disease, together with their families, experience the transition from pediatric care to adult care. The family experience described goes beyond a simple change of environment, configuring itself as a complex process, permeated by issues related to the adolescent’s development, exchange of roles within the family dynamic, continuity of health care, disease prognosis, among others.

Family members and adolescents interviewed in this study described the transition of care as an inevitable and often unwanted process, becoming a challenge to be overcome by the family without having been previously prepared for it. Feelings such as sadness due to the breakdown of emotional ties with pediatric professionals and anxiety arising from the difficulty in establishing relationships of trust with the adult care team were reported by research participants. This finding is supported by other studies^(8,17,18)^, which demonstrate that cultural differences between pediatric and adult care create significant barriers to continuity of care during the transition process.

Both adolescents and family members emphasized the importance of emotional and clinical support from the health team, highlighting that a good relationship with the team favors acceptance of the diagnosis, adherence to treatment, and a better health prognosis. One of the main issues raised in this study was the fear of not being able to establish trusting relationships with the adult team, which could result in loss of health monitoring and a worsening of the clinical condition. The importance of the link with health teams has already been highlighted in several international studies^(1,17,18,19)^, which reinforces the need for the healthcare team to analyze this issue carefully when developing plans for the transition of care.

According to Rosen^(20)^, pediatric care is distinct from adult care due to a functional reason: caregivers play a central role in treatment, and the team’s work must be supportive. This particularity, however, becomes dysfunctional when dealing with older adolescents and young adults, since they must take responsibility for their own care and the development of autonomy must be encouraged. In this context, the roles of caregivers and adolescents must be redefined within family dynamics^(20)^.

This study revealed that the family wants to actively participate in the care transition phase, recognizing the importance of gradually transferring responsibility for treatment and helping the adolescent to achieve autonomy and independence. This transfer from parental care to young adult self-care is a critical event for the entire family and was mentioned both by participants in this study and in previous research on the subject^(1,8,17,21)^.

Although they recognize the need to transfer care within the family dynamic, many family members demonstrate concern and fear in allowing the young person to take control of their treatment. This reluctance stems from a lack of confidence in the young person’s ability to fully assume responsibility for their health, an aspect also addressed in the literature^(17)^. This expectation of family members towards this teenager generates anxiety in a being under identity construction, who has difficulty trusting in his own capabilities and ability to establish links of trust with the adult outpatient health team. At the time of transition of care, the family wants to trust that the adolescent will be able to take full responsibility for treatment alone, and be independent and self-sufficient in his or her decisions^(17)^.

This study revealed that many adolescents are concerned about not knowing their clinical condition well and not being able to convey the necessary information to the adult team doctor, since they will now be attending medical appointments alone. The fear of not adapting to this change in team and how their treatment will be conducted causes anxiety in this young person, a feeling reported in participants of other similar studies^(1,8,17,21)^.

Some factors that would facilitate the care transition process were highlighted by families in this study and corroborated with findings in the literature, such as building bridges between services, improving communication between pediatric and adult teams^(17)^, visiting the adult service, and meeting the new health team before the transition, participating in the preparation of a well-structured transition project along with health professionals , talking to the team in advance about the differences between services, and having the opportunity to establish early links with the adult health team, while still being followed up in pediatrics^(22)^.

Thus, for this research, based on the theoretical framework of Symbolic Interactionism, we consider that the meanings for the care transition process are constructed from the family’s social interactions, whether these interactions are among its members, with the health team, or with other patients. Having a child in the process of transitioning care causes the family to develop meanings and perspectives that arise from social interaction. Studying this family allows us to understand the meanings it attributes to these symbols and their consequent behaviors.

## CONCLUSION

The findings of this study corroborate that the transition of care is a challenging phase for adolescents/young adults and their families, as it necessarily implies a change in roles within the family. This is the moment when caregivers step back from the spotlight, gradually handing over control of the situation to the teenager and assuming the role of supporter. The adolescent, in turn, must assume responsibility for his/her treatment and health care, actively participating in decisions involving his/her health. The involvement of the family and the interdisciplinary team is fundamental in this process and in the success of the care transition, maintaining adherence and quality of treatment.

It is impossible not to emphasize the undeniable importance of the topic, given the current epidemiological scenario, where chronic-degenerative diseases are increasingly gaining prominence and these children are more frequently reaching adolescence and adulthood, being direct beneficiaries of structured plans and teams prepared to lead the transition from pediatrics to adult service, taking into account the particularities that permeate the adolescent phase and its transition to adulthood.

This information highlights the importance of creating public health policies with initiatives aimed at care transition, which must be planned by the services considering the composition of care transition teams and the preparation of an individualized plan for the adolescent and their family, involving them in the construction of this plan and in decision-making, providing shared information, reducing fears and anxiety, as well as acting to stimulate the autonomy of this adolescent and/or young adult in conducting their treatment.

Among the restrictions of the study, the fact that the research was conducted in only one public health service stands out, hindering the comparison of the experiences of patients treated in different places, as well as in private health care institutions. Interviewing patients from different sociocultural and diagnostic contexts, as well as young adults who made the transition some time before, were also limiting factors. It is recommended that future research be conducted approaching different sociocultural contexts to expand knowledge on the topic.

## Data Availability

All the dataset supporting the results of this study is available upon request from the corresponding author [Júlia Rudzinski Roveri]. The dataset is not publicly available due to containing information that compromises the privacy of the research participants.
