# Supplementary material for: Experience of adolescents/young adults with chronic kidney disease and of their families during transition of care
Source: Rev Esc Enferm USP. 2025 Jun 20;59:e20240365. doi: 10.1590/1980-220X-REEUSP-2024-0365en (PMC12184772; doi:10.1590/1980-220X-REEUSP-2024-0365en)
Supplement: Translation

# Translation: Experiência do adolescente/adulto jovem com doença renal crônica e de sua família durante transição de cuidados

**DOI:** 10.1590/1980-220X-REEUSP-2024-0365pt

**Authors:** Júlia Rudzinski Roveri, Maiara Rodrigues dos Santos, Ana Márcia Chiaradia Mendes-Castillo, Aline Oliveira Silveira, Regina Szylit, Maira Deguer Misko

**Affiliations:** 1 Universidade Estadual de Campinas, Faculdade de Enfermagem, Campinas, SP, Brasil.; 2 Universidade de São Paulo, Escola de Enfermagem, Departamento de Enfermagem Materno-Infantil e Psiquiátrica, São Paulo, SP, Brasil.; 3 Universidade de Brasília, Faculdade de Ciências da Saúde, Departamento de Enfermagem, Brasília, DF, Brasil.

**Keywords:** Insuficiência Renal Crônica, Doença Crônica, Transição para Assistência do Adulto, Adolescente, Adulto Jovem, Enfermagem Pediátrica

## Abstract

**Objetivo:**

Compreender experiência do adolescente/adulto jovem com doença renal crônica e de sua família, no processo de transição de cuidados de serviço pediátrico para serviço adulto.

**Método:**

Estudo qualitativo, fundamentado nos pressupostos da Teoria Fundamentada nos Dados e no referencial teórico do Interacionismo Simbólico. Entrevistadas oito famílias de adolescentes/adultos jovens diagnosticados com doença renal crônica, em processo de transição do cuidado, acompanhados em um hospital público de ensino, durante os anos de 2019 e 2020. Dados foram obtidos por meio da observação participante, entrevistas semiestruturadas e consultas a prontuários clínicos.

**Resultados:**

A análise comparativa dos dados permitiu desvendar experiência da família no processo, representada pelas categorias “tendo que avançar de fase”, na qual os participantes descrevem a necessidade de fortalecer a independência do adolescente/adulto jovem a partir da mudança de papéis dentro da família e “desbravando o novo mundo”, onde a autonomia do adolescente/adulto jovem deve ser estimulada.

**Conclusão:**

Estudo destacou importância de planos individualizados para transição, colaboração entre família e equipe de saúde, e fortalecimento da independência e autocuidado do adolescente e adulto jovem.

## INTRODUÇÃO

A prevalência de doenças crônicas na adolescência tem crescido consideravelmente nas últimas décadas. É estimado que aproximadamente 10% dos adolescentes de países ocidentais convivam com condições crônicas de saúde nos dias de hoje^(1)^. O contínuo progresso tecnológico na área médica tem contribuído para melhores prognósticos, permitindo que esses jovens atinjam a idade adulta, aumentando a necessidade de realizar a transição dos cuidados pediátricos para o sistema de saúde voltado para adultos^(2,3)^.

O conceito mais utilizado na literatura para o termo “transição de cuidados” é o da Society for Adolescent Medicine, sendo definido como “um movimento intencional e planejado de adolescentes e jovens adultos com condições crônicas de um serviço pediátrico para serviços de saúde voltados para adultos”^(4,5)^. O adolescente/jovem adulto que vive com a doença crônica, além das mudanças esperadas para a idade, deverá enfrentar também alterações em seu cuidado de saúde e na forma como ele será oferecido, acrescentando complexidade a um período de transição já conturbado^(6,7)^.

Embora diversas recomendações tenham sido feitas nos últimos anos, a transição para o cuidado adulto ainda representa um desafio para adolescentes, trabalhadores da área da saúde e familiares. Esse processo exige uma alteração na dinâmica familiar, em que novos papéis e responsabilidades são assumidos entre seus membros^(8)^. Muitos adolescentes vivenciam a transição de forma desarticulada, enfrentando dificuldades tanto em deixar o ambiente pediátrico familiar quanto na adaptação no ambiente adulto. Profissionais pediátricos mostram resistência em transferir a responsabilidade para os pacientes, enquanto a equipe de saúde adulta nem sempre está preparada para lidar com jovens^(1)^.

A transição para o cuidado adulto deve considerar a complexidade da doença, o contexto familiar e a prontidão do adolescente para assumir a responsabilidade por sua saúde, em vez de se basear apenas na idade cronológica^(9)^. Uma transição abrupta, sem planejamento ou coordenação, pode causar desamparo, comprometer a adesão ao tratamento e agravar o quadro clínico. Por isso, é essencial que a equipe de saúde elabore um plano gradual e estruturado para assegurar a continuidade do cuidado, promovendo uma melhor adesão ao tratamento e prevenindo complicações futuras da doença crônica^(6,7)^.

Para que isso seja possível, entende-se que é de extrema importância conhecer os sentimentos e expectativas do adolescente e de sua família que irão passar para o cuidado adulto em breve, assim como a experiência vivenciada pelo jovem adulto que já está sendo acompanhado no ambulatório adulto e sua família. Esperamos que esse conhecimento permita ao profissional da saúde traçar um programa de transição de cuidados individualizado e organizado, levando em consideração a dinâmica familiar, a doença e o cenário sociocultural em que o adolescente está inserido^(10,11)^.

Desse modo, esse estudo teve como objetivo conhecer a experiência da família e/ou do adolescente/jovem adulto com doença renal crônica durante seu processo de transição de cuidados da pediatria para um serviço adulto.

## MÉTODO

### Desenho do Estudo

Estudo qualitativo, apoiado nos pressupostos do referencial metodológico da Teoria Fundamentada nos Dados, desenvolvido a partir da ótica do Interacionismo Simbólico como referencial teórico. Essa metodologia foi escolhida por sua capacidade de orientar a coleta, organização e análise dos dados, com o objetivo de desenvolver uma teoria, hipótese ou conceito baseado nas informações obtidas durante a pesquisa. Ela busca extrair significados das experiências vivenciadas, com ênfase nas interações sociais. Além disso, permite a construção de categorias inter-relacionadas que contribuem para a formação de uma estrutura teórica, ampliando a compreensão dos temas estudados, explorando novas perspectivas e esclarecendo as experiências analisadas^(12)^. As recomendações do guia Consolidated Criteria for Reporting Qualitative Research (COREQ)^(13)^ nortearam a construção do presente trabalho.

### Local

A coleta de dados foi realizada no Ambulatório de Nefrologia Pediátrica e no Ambulatório de Nefrologia Adulta, ambos pertencentes ao Centro Integrado de Nefrologia (CIN), unidade que integra um complexo hospitalar público de nível terciário, voltado para atividades de ensino e pesquisa, localizado no interior do estado de São Paulo. Essa unidade é exclusiva para cuidados de pacientes com doença renal crônica, sendo 30% dos pacientes pediátricos e 70% adultos. O acompanhamento ambulatorial abrange todas as modalidades terapêuticas e oferece atendimento para todos os estágios de doenças renais, desde o tratamento conservador até o transplante de rim. A equipe médica determina quais adolescentes devem realizar a transição de cuidados para o serviço adulto, utilizando como principais critérios a idade de dezoito anos e a estabilidade clínica do adolescente. Pacientes com o quadro de saúde instável e famílias com significativa vulnerabilidade social ou emocional podem ter a transição de cuidados adiada, até a estabilidade clínica ou familiar, o que pode ocorrer após os 20 anos de idade do paciente.

### População

Os participantes desta pesquisa incluíram adolescentes (de 12 a 18 anos) e adultos jovens (de 19 a 24 anos) com doença renal crônica, bem como seus familiares. A escolha dessa faixa etária visou abranger a experiência completa da transição de cuidados, considerando todos os estágios da adolescência de forma equânime. O adolescente ainda em acompanhamento pediátrico pode compartilhar suas expectativas para o futuro, enquanto o adulto jovem, já atendido em ambulatório adulto, expressa seus sentimentos sobre a transição já vivenciada. Vale ressaltar que todos os participantes adolescentes e adultos jovens realizavam tratamento conservador no ambulatório, com exceção dos membros das famílias 4 e 5, que já haviam passado por transplante renal.

Atualmente, a definição de família varia de acordo com o referencial de cada indivíduo, considerando seus valores e crenças. Ou seja, família é definida pelos seus próprios membros, independente de laços de consanguinidade. Porém, o sistema familiar não é apenas composto por pessoas que possuem vínculos afetivos, mas também por indivíduos que desempenham uma função cultural e social dentro deste sistema^(14)^, e esta foi a definição utilizada na construção deste trabalho.

Foram seguidos os seguintes critérios de inclusão: ser familiar de adolescente ou jovem adulto com doença renal crônica que vivenciou ou irá vivenciar o processo de transição de cuidados no CIN; ser adolescente ou jovem adulto diagnosticado com doença renal crônica que vivenciou ou irá vivenciar o processo de transição de cuidados no CIN. Como critérios de exclusão, foram considerados: familiares, adolescentes e jovens adultos com déficit neurológico ou de fala, impossibilitando a execução das entrevistas; adolescentes ou jovens adultos que estivessem hospitalizados ao longo da fase de obtenção dos dados.

Desta maneira, participaram da pesquisa 08 famílias, com total de 14 membros familiares entrevistados: seis adolescentes e um adulto jovem com doença renal crônica, quatro mães, dois pais e uma irmã. Destaca-se que o jovem adulto da família 05 não participou da pesquisa devido a atraso no desenvolvimento neuropsicomotor. No caso da família 06, apenas o jovem adulto participou da pesquisa porque ele não frequenta mais o serviço com acompanhante. Nenhuma das famílias convidadas a participar do estudo apresentou recusa. Os participantes estão descritos no Quadro 1.

**Quadro 1:** Caracterização dos participantes da pesquisa – Campinas, SP, Brasil, 2020.

Grupo Amostral	Família	Nome	Idade	Parentesco	Diagnóstico	Tempo de transição
1 – Ambulatório de Pediatria	Família 1	Rosana	42	Mãe	Síndrome Nefrótica	Sem previsão
		Marcela	17	Paciente		
	Família 2	Clara	45	Mãe	Síndrome Nefrótica	Última consulta pediatria
		Eduardo	18	Paciente		
	Família 3	André	43	Pai	Glomerulopatia	Última consulta pediatria
		Laís	19	Paciente		
	Família 4	Mara	48	Mãe	Cistinose Nefropática	Sem previsão
		Vitor	20	Paciente		
2 – Ambulatório Adulto	Família 5	Francisco	40	Pai	Insuficiência Renal Crônica	1 mês
	Família 6	Antônio	18	Paciente	Síndrome Nefrótica	5 meses
	Família 7	Paula	46	Mãe	Insuficiência Renal Crônica	5 meses
		Valéria	18	Paciente		
	Família 8	Milena	25	Irmã	Insuficiência Renal Crônica	1^a^consulta
		Caio	18	Paciente		

### Definição da Amostra

A obtenção de dados foi concluída ao alcançar a saturação teórica, etapa em que a recorrência e a falta de novos elementos indicam que o aprofundamento da teoria foi suficiente. Esse conceito é amplamente utilizado em pesquisas qualitativas na área da saúde. Assim, o número de participantes não foi pré-definido, sendo determinado conforme os dados eram coletados e analisados^(15)^.

O grupo amostral intitulado “Ambulatório de Pediatria” foi constituído por quatro famílias que estavam em acompanhamento no ambulatório de nefrologia pediátrica, mas que já haviam sido comunicados pela equipe de saúde que o processo de transição para o cuidado adulto estava próximo de se concretizar. Esse primeiro momento teve como objetivo apreender como o adolescente/adulto jovem e sua família vivenciavam a experiência da transição de cuidado enquanto ainda era uma possibilidade, quais eram seus sentimentos e expectativas frente a isso. O segundo grupo amostral foi composto por quatro famílias que já tinham vivenciado o processo de transição para o cuidado adulto e estavam iniciando ou já estavam em acompanhamento no ambulatório adulto, o que foi fundamental para ampliar o olhar para a experiência da família frente ao processo de transição. É importante ressaltar que os grupos amostrais são distintos e não se sobrepuseram; ou seja, as famílias do primeiro grupo amostral não participaram da pesquisa no segundo momento e vice-versa.

### Coleta de Dados

A coleta de dados foi realizada no período de agosto de 2019 a março de 2020, utilizando os métodos de observação participante, entrevistas semiestruturadas e consultas aos prontuários.

A observação participante constitui-se em uma estratégia que possibilita ao pesquisador imergir na situação investigada, acompanhando as expressões verbais e não verbais, além de contribuir para a identificação de detalhes que, até então, não haviam sido percebidos ou relatados durante as entrevistas. A observação foi realizada tanto na sala de espera quanto durante as consultas de enfermagem que precedem as consultas médicas. Cada família foi acompanhada, em média, por 60 minutos. Essas observações foram anotadas em um diário de campo no momento em que eram feitas, sendo denominadas como “notas de observação”.

A entrevista semiestruturada foi conduzida pela pesquisadora responsável, sendo composta por duas seções: a primeira destinada à obtenção de informações sociodemográficas e de caracterização da família e a segunda composta pela questão norteadora “Como tem sido/foi para você vivenciar com seu filho/familiar o processo de transição de cuidados de um serviço pediátrico para um serviço adulto?” Os discursos decorrentes dessa questão foram permeados por novos questionamentos, como por exemplo, “Como esta situação fez você se sentir?” e “O que você acredita que poderia ter lhe ajudado nesta situação/momento?”, a fim de aprofundar a descrição da experiência vivenciada. As entrevistas tiveram duração média de 30 a 40 minutos e ocorreram uma única vez com cada participante.

O contato com os participantes aconteceu na área de espera do ambulatório pediátrico ou adulto, enquanto aguardavam o atendimento médico ou de enfermagem. A pesquisadora foi apresentada à família pelos profissionais do ambulatório, não tendo estabelecido nenhum vínculo ou contato prévio com essas famílias, seja antes ou após a entrevista. Apenas a pesquisadora coletou os dados, e esta não possui qualquer vínculo com o local de estudo, o que ajudou a minimizar possíveis desconfortos ou vieses no acesso aos sujeitos da pesquisa.

A entrevista com cada família foi realizada individualmente, respeitando-se a privacidade do entrevistado, no local e no momento de preferência da família, sendo que todos os entrevistados optaram por realizar a entrevista no próprio ambulatório em que eram atendidos, em sala privativa e previamente reservada pela pesquisadora para este fim. Foi dada à família a opção de as entrevistas serem realizadas sem a presença do adolescente/adulto jovem e o inverso também foi realizado, contudo nenhuma família utilizou tal recurso.

Os adolescentes foram convidados a participar da entrevista, podendo escolher entre comparecer acompanhados por seus familiares ou de forma individual. Todos decidiram participar junto com seus familiares e expressaram-se abertamente, compartilhando seus sentimentos e expectativas. Os participantes foram nomeados com pseudônimos, assegurando o sigilo de suas identidades.

A pesquisadora principal conduziu a coleta de dados de maneira completa e, para isso, passou por treinamento para realizar entrevistas com famílias, oferecido pelo grupo de pesquisa ao qual pertence. As entrevistas foram registradas em dispositivo digital e, em seguida, transcritas na totalidade.

As transcrições foram revisadas por membros do grupo de pesquisa e os participantes não tiveram acesso às transcrições.

Também foram realizadas consultas nos prontuários físicos e eletrônicos dos pacientes, com o objetivo de obter informações como diagnóstico, tempo de acompanhamento no serviço, tratamento e prognóstico da doença.

### Análise e Tratamento dos Dados

Os depoimentos gravados foram transcritos na íntegra de maneira manual e analisados segundo o método comparativo contínuo, por meio do qual as categorias foram confrontadas para identificar conceitos semelhantes ou diferentes, simultaneamente à coleta de dados. As categorias foram revisadas pelos membros da equipe.

A análise dos dados foi conduzida com base nos princípios da Teoria Fundamentada nos Dados, utilizando as codificações aberta, axial e seletiva. Assim, a primeira etapa do processo analítico foi a codificação aberta, que consiste na leitura atenta do pesquisador de cada frase dita pelo entrevistado, seguida por reflexão e conceptualização da frase. Nessa etapa, foram atribuídas expressões aos fragmentos da entrevista, com o objetivo de formar códigos. Esse processo envolveu a análise de frase por frase e palavra por palavra da entrevista, considerando as perguntas e os objetivos da pesquisa^(16)^. À medida que as perguntas foram sendo respondidas, foi possível identificar as primeiras propriedades, que possibilitaram a formação das categorias apresentadas neste trabalho.

A codificação axial foi a segunda fase da análise, caracterizada pelo processo de vincular categorias às suas subcategorias, com o objetivo de gerar explicações mais detalhadas e completas sobre os fenômenos. Como as conexões entre categorias podem ser bastante implícitas, Strauss e Corbin^(16)^ sugerem a elaboração de um esquema que possa ser utilizado para estruturar as relações emergentes. Neste estudo, foi adotado o tipo denominado “processo” para a codificação axial. Strauss e Corbin^(16)^ o definem como uma série de sequências evolutivas de ação/interação que acontecem ao longo do tempo e do espaço, alterando-se ou permanecendo constantes em determinados momentos, em resposta à situação ou ao contexto. Assim, o processo nos dados é representado por eventos e ocorrências que podem ou não se desenrolar em sequências contínuas.

A codificação seletiva é a terceira fase da análise, na qual se procurou integrar e aprimorar a teoria. Assim como nas etapas anteriores da análise, essa integração envolve uma interação entre o analista e os dados, pois abrange a evolução do pensamento ao longo do tempo, por meio da imersão nos dados e do conjunto de resultados obtidos.

Os dados foram então comparados na busca por similaridades e diferenças. Esse processo permitiu analisar detalhadamente como as palavras e frases foram utilizadas, associando-as a sentimentos e experiências, auxiliando assim na interpretação dos dados, formando agrupamentos de conceitos mais abstratos, que são denominados de categorias e são exemplificados no Quadro 2.

**Quadro 2:** Exemplo de Categorização – Campinas, SP, Brasil, 2020.

Códigos	Categoria
– Ficando um pouco na expectativa. – Tendo um pouco de ansiedade. – Sabendo que não são os mesmo profissionais, mas acreditando que vai ter o mesmo encorajamento. – Acreditando que vai agregar mais. – Recebendo a notícia que ia para o ambulatório adulto. – Ficando na expectativa com a transição.	TENDO QUE AVANÇAR DE FASE

### Aspectos Éticos

O projeto foi encaminhado ao Comitê de Ética em Pesquisa da Universidade Estadual de Campinas, sendo aprovado sob o parecer de número 3.276.133. Cabe ressaltar que os aspectos da Resolução 466/2012, do Conselho Nacional de Saúde, foram rigorosamente respeitados. Todos os participantes foram informados sobre os objetivos do estudo, os procedimentos e métodos, especialmente em relação ao uso do gravador durante as entrevistas, à garantia de confidencialidade das informações, ao direito ao anonimato e à liberdade de optar por participar ou não da pesquisa. Os adolescentes e seus familiares foram convidados a integrar o estudo e, após a leitura do Termo de Assentimento Livre e Esclarecido (para participantes menores de 18 anos) e Termo de Consentimento Livre e Esclarecido (para maiores de 18 anos e todos os familiares), dúvidas foram esclarecidas e a assinatura dos termos pelos envolvidos foi solicitada.

## RESULTADOS

Os significados conferidos pelos familiares, para a experiência de vivenciar o processo de transição para o cuidado adulto, foram entendidos após a avaliação e a comparação contínua dos dados. Assim, o processo que reflete a experiência familiar é composto por duas categorias: “Tendo que avançar de fase” e “Desbravando o novo mundo”.

### Tendo Que Avançar de Fase

A experiência da família de adolescentes e/ou adultos jovens com doença renal crônica durante o processo de transição de cuidados está intimamente ligada ao processo de adaptação familiar, particularmente quando o diagnóstico é feito ainda na infância. Esse processo de adaptação envolve uma combinação de fatores emocionais e práticos, refletindo tanto as mudanças na vida cotidiana da família quanto a gestão das emoções diante de uma condição crônica e potencialmente debilitante.

Muitas famílias relataram uma diminuição da fragilidade emocional ao iniciarem o acompanhamento no serviço pediátrico. Esse suporte da equipe de saúde contribuiu para o enfrentamento da doença e para o estabelecimento de uma rotina de tratamento. O convívio no ambulatório estreitou os vínculos de afeto entre familiares e trabalhadores de saúde, diminuindo a carga emocional que a doença carrega.

*“Tem o lado pessoal junto aqui. Porque tem 16 anos que venho aqui, então eles me conhecem desde pequeno.” [Eduardo, 18 anos.]*

*“Todas as enfermeiras têm amizade com ele, todos conhecem ele desde quando ele era criança. ‘Ô Joãozinho, você já está grande, vai passar no adulto agora! ’. Já fica assim: ‘Nossa, você vai passar no adulto? Não, não pode ser!’” [Milena, irmã]*

*“Marcela aguarda na sala de espera juntamente com sua mãe, enquanto olha o celular. É chamada pela enfermeira e entra no consultório de enfermagem sozinha. Dentro do consultório, Marcela já sabe o procedimento: tira o sapato e a blusa amarrada na cintura. Sobe na balança e a enfermeira, enquanto anota seu peso, pergunta sobre o namorado, com quem Marcela começou a se relacionar recentemente. Marcela começa a contar sobre seu último final de semana e a conversa flui como duas amigas.”[anotação de campo realizada pela pesquisadora a partir da observação participante]*

Durante a transição de cuidados, a família romperá vínculos afetivos. Ao sair da pediatria, a família estará deixando para trás anos de interações sociais construídas, experiências e aprendizados compartilhados, momentos tristes e felizes que compuseram suas trajetórias, para adentrar em um mundo com novos significados. As famílias relatam insegurança devido à ausência de suporte e informações, além de medo e tristeza em avançar de fase, especialmente quando não se tem as instruções e regras necessárias para enfrentar essa mudança.

*“Eu não quero ir* (para o ambulatório adulto)*. Eu fico triste, eu não quero ir. Eu nem fico pensando muito, não quero ir para lá não, eu gosto daqui.” [Marcela, 17 anos.]*

*“Só o medo. Não sei como é lá, não sei como vai ser. Só medo.” [Laís, 19 anos]*

*“A junta médica do lado do infantil são muito bonzinho, tem umas doutora ali, nossa, muito boa. Se eu pudesse, eu deixava aqui (no ambulatório infantil).Você vê que é mais carinhoso, assim. Não gostei muito deles, no adulto lá, que não deram muita atenção”. [Francisco, pai]*

A possibilidade de mudanças no tratamento e nas rotinas de saúde traz para a família o sentimento de que o processo de transição de cuidados pode acarretar um risco à continuidade do tratamento, ou agregar novas restrições para a vida cotidiana, como é o caso da hemodiálise.

*“Ele conversa muito sobre isso: ‘Vai ter muita diferença?’ (...) Será que eu vou mudar algum medicamento? Será que eu vou mudar alguma rotina?’ Ele ainda não sabe.” [Milena, irmã.] “Acho que meu maior medo é chegar lá e, sabe, a hemodiálise. Porque aqui, não sei como que é lá, mas aqui eles dão remédio, porque o cuidado com a criança é diferente que com um adulto. Eles procuram dar a privacidade para criança continuar crescendo, mas lá não.” [Laís, 19 anos.]*

Não apenas os procedimentos e as rotinas são diferentes no ambulatório adulto, mas também o espaço físico e as interações pessoais no novo ambiente, o que gera um impacto significativo na família. Isso ocorre porque as relações tendem a ser mais distantes e as consultas mais protocolares. Muitas famílias relatam despreparo para enfrentar essa mudança de equipe e de ambulatório, ao mesmo tempo em que se sentem pressionadas a avançar de fase e aceitar a transição.

O jovem adulto e sua família terão que interagir com uma nova e desconhecida equipe de saúde, com quem deverão estabelecer relações de confiança semelhantes àquelas vividas no ambiente pediátrico. No entanto, nas famílias, existe o receio de que essas novas relações se tornem mais difíceis e rígidas. Além disso, a dificuldade natural de interação típica da adolescência, agravada pela presença de uma doença crônica, é uma preocupação frequentemente expressa pelas famílias que vivenciam esse processo.

*“Você já conhece (a pediatria), já sabem quem você é, já sabem do seu jeito. Uma hora você está alegre, outra hora você está desanimado. Então parece que você já se torna amigo ali. Do outro lado (ambulatório adulto) eu já fui várias vezes, mas é tudo meio seco.” [Mara, mãe]*

*“Eu penso que o médico (do ambulatório adulto) não vai tratar você igual. Talvez ele seja um pouco mais rígido, não pergunte seu pessoal.” [Eduardo, 18 anos.]*

*“No começo eu estranhei um pouquinho, achei um pouquinho diferente. Aqui (pediatria) eles faziam de um jeito, lá já é de outro. O modo de pesar é diferente.” Antônio, 18 anos.*

Esse processo gera ansiedade e sofrimento nas famílias, que se dão conta neste momento de que terão que desenvolver novas habilidades para que o adolescente ou adulto jovem ganhe voz e seja o narrador de sua história. O medo de não estabelecer vínculos com a nova equipe está profundamente relacionado ao receio de se apresentar a um grupo até então desconhecido.

*“Ah, é que geralmente sempre minha mãe fala por mim. Agora estou tentando falar sozinha, agora. Isso que é meio estranho.” [Valéria, 18 anos.]*

As famílias temem não conseguir replicar as condutas e tratamentos realizados durante o acompanhamento na nefropediatria para a equipe adulta, o que poderia comprometer o seguimento e a saúde do adolescente. A continuidade do tratamento parece estar em risco, e elas sentem que terão que enfrentar essa jornada sozinhas e com poucas informações.

*“Quanto à transição (…) eu não sou médico para dizer, pra contar minha história assim, sobre o que aconteceu, né? Eu sei que aconteceu, mas a parte técnica eu não vou saber falar. Dizer: ‘ele começou com síndrome nefrótica, aconteceu tudo isso, ele tem um caso assim’, contar sobre o meu caso, sabe? E já deixar pronto para o médico pegar e falar: ‘ah, esse daqui é o Eduardo!’. Já saber quem é o Eduardo. Me deixaria um pouco mais tranquilo, porque ele já saberia a minha história um pouco. Claro que não saberia tudo, porque está começando agora o acompanhamento.” [Eduardo, 18 anos.]*

Na categoria “Tendo que Avançar de Fase”, a família enfrenta a inevitável transição de cuidados, buscando construir uma nova identidade enquanto interage com pessoas e situações desconhecidas. Ao longo desse processo, a família percebe a necessidade de apoiar a independência do adolescente, e buscando firmar nossos símbolos a partir das novas relações que começam a ser estabelecidas, garantindo assim a continuidade dos cuidados de saúde. Dito isso, não há como recuar e os adolescentes e/adultos jovens e suas famílias passam a viver um novo momento, a partir da transição de cuidado, representada pela segunda categoria, nomeada “Desbravando o novo mundo”.

### Desbravando o Novo Mundo

A transição para o cuidado adulto representa o início de uma nova era na vida da família e configura-se como um dos maiores obstáculos desde o diagnóstico. A categoria “Desbravando o Novo Mundo” representa o esforço da família em reunir as ferramentas indispensáveis para se adaptar a um contexto diferente, caracterizado por novas estruturas, regras e rotinas. Esse período também se destaca pela reorganização dos papéis familiares, que devem se ajustar às demandas dessa nova realidade.

Com o diagnóstico da criança, a família reorganiza-se para que um de seus membros, geralmente a mãe, assuma a centralização dos cuidados e dedique-se quase exclusivamente ao filho, supervisionando o tratamento e o acompanhamento da saúde. Nesse processo, essas mães passam a ocupar um novo papel na dinâmica familiar: o de cuidadora de um filho com doença crônica. Essa configuração costuma perdurar ao longo da infância, mas, na transição para a vida adulta, muitas mães retomam suas atividades profissionais e precisam transferir gradualmente o controle e as responsabilidades do tratamento para os filhos, agora adolescentes, desafiando-os a assumir a responsabilidade pela própria condição clínica.

*“Faz dois meses, agora que eu voltei à rotina normal, porque até então...antes de ele ficar doente, eu trabalhava, depois eu não...eu fazia alguma coisa assim, pra preencher o tempo (...) mas assim, agora estou trabalhando mesmo.” [Clara, mãe]*

*“(A mãe) controla tudo, deixa os remedinhos cortados no miligrama certo, ela fica no pé do horário (...) não sei se é a fase da adolescência, mas ele é muito relutante. Então, tem que ficar em cima.” [Milena, irmã.]*

O adolescente, ao entrar na vida adulta, ganha responsabilidades e autonomia, embora nem sempre venham acompanhadas da maturidade emocional ou prontidão necessárias para assumir o controle da situação. A transição de cuidados exige que ele se responsabilize pela própria doença e tratamento, assim como pelas consequências decorrentes da não adesão ao tratamento. Nesta etapa, a família frequentemente se questiona sobre a prontidão do adolescente para assumir um papel mais ativo em seu cuidado, ao mesmo tempo em que teme possíveis agravamentos no quadro clínico. Muitos familiares sentem-se inseguros e até ameaçados pela possibilidade de perder o controle sobre a saúde do adolescente, o que pode gerar preocupações quanto à continuidade do tratamento e ao risco de piora na condição clínica.

*“É estranho eu deixá-la vir sozinha. Porque o dia que ela falou: ‘mãe, eu vou para o adulto e a senhora não pode ir!’ Mas espera aí, quanto tempo eu acompanhei? Eu cuidei, remédio, e tal. ‘Mas mãe, eu estou com 18, está na hora de cortar o cordão, né?” [Paula, mãe].*

*“Hoje ele vai sentar numa sala com um médico, que na primeira vez eu vou com ele, mas pode ser que nas próximas eu não entre mais, então eu vou ter que acreditar que tudo o que o médico falar pra ele, ele vai ter que passar pra mim ou pra minha mãe.” [Milena, irmã].*

*“Geralmente minha mãe fala por mim. Agora estou tentando falar sozinha. Isso é meio estranho.” [Valéria, 18 anos.]*

O ambulatório adulto geralmente é descrito pelos familiares como “a vida real”, enquanto a pediatria apresenta-se como um mundo “de faz de conta”. Esses familiares trazem consigo a angústia de ver que seus jovens cresceram e estão entrando no mundo real e tendo que assumir o controle de sua doença.

*“Aí é uma nova fase de vida para ela, que eu acho que é mais difícil. Ela se adaptar, a adaptação dela lá. Porque ela vai ter que lidar com pessoas, acabou aquele negócio de criança, pré- adolescente. A coisa para adulto é mais séria, em todos os fatores né, não só aqui.” [Rosana, mãe.]*

Nesta mudança de papéis, os familiares deixam o protagonismo do cuidado e assumem a função de apoiadores, enquanto o adolescente volta-se para si em busca de instrumentos e recursos para desenvolver autonomia e enfrentar o medo de não se sentir preparado para este momento. No ambiente adulto, os familiares serão vistos como um suporte, mas será o jovem adulto que deverá responder pelas suas decisões e comportamentos frente à doença. Referem o receio e medo pela cobrança que imaginam que passarão a receber, quando o processo de transição de cuidado do serviço de pediatria para o adulto de fato concretizar-se.

*“Aqui (pediatria) eu era meio que: ‘Tá bom, você não vai ter o cargo, ainda. O peso não vai pra tu, vai pro seu acompanhante’. Mas agora o peso vai pra mim. Alguma coisa, vai ser a culpa minha.” [Caio, 18 anos.]*

*“A partir do momento que ela fizer 18 anos, vai mudar muita coisa na vida dela. Ela vai ter que encarar as coisas de frente, deixou de ser criança, deixou de ter uma certa regalia aqui. A coisa é mais séria.” [Rosana, mãe.]*

*“Agora a responsabilidade pesa sobre ele. Quando a gente acompanha, quando a gente tá perto é mais fácil, porque vem pra gente, a gente cobra e fica em cima. Agora, quando é pra ele, pra ele pegar (...) eu sinto por ele, que agora ele vê que a responsabilidade é dele. Não é toda vez que a gente vai poder entrar, que a gente vai poder acompanhar, que agora a responsabilidade é dele. Então um pouco vai ser bom para o amadurecimento.” [Milena, irmã.]*

Este processo analítico explora, na experiência da família, a dinâmica entre enfrentar a inevitável transição para o ambulatório de cuidados adultos e lidar com o rompimento dos laços estabelecidos no serviço pediátrico. Esse rompimento é acompanhado do temor pelas possíveis consequências, como a descontinuidade do tratamento e o agravamento do quadro clínico do adolescente. Nesse contexto, o adolescente ou adulto jovem emerge como protagonista de seu tratamento, buscando maior autonomia e independência.

## DISCUSSÃO

Os resultados obtidos neste estudo ampliam a compreensão acerca de como adolescentes e adultos jovens com doença renal crônica, juntamente com seus familiares, vivenciam a transição do cuidado pediátrico para o cuidado adulto. A experiência familiar descrita transcende uma simples mudança de ambiente, configurando-se como um processo complexo, permeado por questões relacionadas ao desenvolvimento do adolescente, troca de papéis dentro da dinâmica familiar, continuidade do cuidado de saúde, prognóstico da doença, entre outros.

Familiares e adolescentes entrevistados neste estudo descreveram a transição de cuidados como um processo inevitável e muitas vezes indesejado, tornando-se um desafio a ser superado pela família sem que tenham sido preparados previamente para isso. Sentimentos como tristeza pela quebra de vínculos afetivos com os profissionais da pediatria e ansiedade advinda da dificuldade em estabelecer relações de confiança com a equipe de cuidados adulta foram relatados pelos participantes da pesquisa. Esta descoberta é apoiada por outros estudos^(8,17,18)^, que demonstram que as diferenças culturais entre os cuidados pediátricos e adultos criam barreiras significativas à continuidade dos cuidados durante o processo de transição.

Tanto os adolescentes quanto os familiares enfatizaram a importância do suporte emocional e clínico da equipe de saúde, destacando que um bom relacionamento com a equipe favorece a aceitação do diagnóstico, a adesão ao tratamento e um melhor prognóstico de saúde. Uma das questões principais levantadas neste estudo foi o receio de não conseguirem estabelecer relações de confiança com a equipe adulta, o que poderia resultar em perda do acompanhamento de saúde e uma piora no quadro clínico. A importância do vínculo com as equipes de saúde já foi destacada em diversos estudos internacionais^(1,17,18,19)^, o que reforça a necessidade de a equipe de saúde analisar essa questão de forma criteriosa ao elaborar planos para a transição de cuidados.

Segundo Rosen^(20)^, o cuidado pediátrico é distinto do cuidado adulto por uma questão funcional: os cuidadores desempenham papel central no tratamento, e o trabalho da equipe deve ser de suporte e apoio. Essa particularidade, no entanto, torna-se disfuncional quando lidamos com adolescentes mais velhos e jovens adultos, uma vez que eles devem se responsabilizar pelos próprios cuidados e o desenvolvimento da autonomia deve ser estimulado. Nesse contexto, os papéis dos cuidadores e dos adolescentes devem ser redefinidos dentro da dinâmica familiar^(20)^.

Este estudo revelou que a família deseja participar ativamente da fase da transição de cuidados, reconhecendo a importância de transferir gradualmente a responsabilidade pelo tratamento e auxiliando o adolescente na conquista da autonomia e independência. Essa transferência do cuidado parental para o autocuidado do jovem adulto é um evento crítico para toda a família e foi mencionado tanto pelos participantes deste estudo quanto em pesquisas anteriores sobre o assunto^(1,8,17,21)^.

Embora reconheçam a necessidade de transferência dos cuidados dentro da dinâmica familiar, muitos familiares demonstram preocupação e medo em permitir que o jovem assuma o controle de seu tratamento. Essa relutância decorre da falta de confiança na capacidade do jovem de assumir integralmente a responsabilidade por sua saúde, um aspecto também abordado na literatura^(17)^. Essa expectativa dos familiares para com esse adolescente gera ansiedade em um ser em construção identitária, que tem dificuldade em confiar nas próprias capacidades e na habilidade de estabelecer vínculos de confiança com a equipe de saúde do ambulatório adulto. No momento da transição de cuidados, a família quer confiar que o adolescente será capaz de assumir total responsabilidade pelo tratamento sozinho, e ser independente e autossuficiente em suas decisões^(17)^.

Esse estudo revelou que muitos adolescentes se preocupam com o fato de não conhecerem bem sua condição clínica e de não serem capazes de transmitir as informações necessárias para o médico da equipe adulta, uma vez que agora eles participarão sozinhos das consultas médicas. O receio de não se adaptarem a essa mudança de equipe e de como seu tratamento será conduzido causa ansiedade nesse jovem, sentimento relatado em participantes de outros estudos semelhantes^(1,8,17,21)^.

Alguns fatores que facilitariam o processo de transição de cuidados foram apontados pelas famílias nesse estudo e corroborados por achados na literatura, tais como construir pontes entre os serviços, melhorando a comunicação entre as equipes pediátrica e adulta^(17)^, visitar o serviço adulto e conhecer a nova equipe de saúde antes da transição, participar da elaboração de um projeto de transição bem estruturado juntamente com os profissionais de saúde, conversar previamente com a equipe sobre as diferenças entre os serviços e ter a oportunidade de estabelecer vínculos precoces com a equipe de saúde do adulto, enquanto ainda acompanham na pediatria^(22)^.

Dessa forma, para esta pesquisa, a partir do referencial teórico do Interacionismo Simbólico, consideramos que os significados para o processo de transição de cuidados são construídos a partir das interações sociais da família, sejam estas interações entre seus membros, com a equipe de saúde, ou com outros pacientes. Ter um filho em processo de transição de cuidados faz com que a família elabore significados e perspectivas que surgem a partir da interação social. Estudar essa família permite conhecer os significados que ela atribui para esses símbolos e seus consequentes comportamentos.

## CONCLUSÃO

As descobertas deste estudo corroboram que a transição de cuidados é uma fase desafiadora para adolescentes/adultos jovens e seus familiares, uma vez que implica necessariamente em mudança de papéis dentro da família. É o momento em que os cuidadores deixam o protagonismo, passando gradualmente o controle da situação para o adolescente e assumindo a posição de apoiadores. O adolescente, por sua vez, deve assumir a responsabilidade pelo seu tratamento e cuidados de saúde, participando ativamente nas decisões que envolvem sua saúde. O envolvimento da família e da equipe interdisciplinar é fundamental nesse processo e no sucesso da transição do cuidado, mantendo a adesão e qualidade do tratamento.

Não se pode deixar de ressaltar a inegável importância do tema, visto o cenário epidemiológico atual, onde as doenças crônico-degenerativas ganham cada vez mais destaque e com maior frequência essas crianças atingem a adolescência e idade adulta, sendo beneficiárias diretas de planos estruturados e equipes preparadas para conduzir a transição da pediatria para serviço adulto, atendendo as particularidades que permeiam a fase da adolescência e sua passagem para a idade adulta.

Essas informações destacam a importância da criação de políticas públicas de saúde com iniciativas direcionadas para a transição de cuidados, que devem ser planejados pelos serviços levando-se em consideração a composição de equipes de transição de cuidados e a elaboração de um plano individualizado para o adolescente e sua família, envolvendo-os na construção deste e nas tomadas de decisões, proporcionando informação compartilhada, redução de medos e ansiedade, bem como atuando para estimular a autonomia deste adolescente e/ou adulto jovem na condução de seu tratamento.

Entre as restrições do estudo, destaca-se o fato de que a pesquisa foi conduzida em apenas um serviço público de saúde, não sendo possível comparar as experiências de pacientes atendidos em locais distintos, bem como em instituições privadas de assistência à saúde. Entrevistar pacientes de diferentes contextos socioculturais e diagnósticos, além de jovens adultos que realizaram a transição há mais tempo, também foram fatores limitantes. Recomenda-se que futuras pesquisas sejam conduzidas abordando diferentes contextos socioculturais para ampliar o conhecimento sobre o tema.

## Data Availability

Todo o conjunto de dados que dá suporte aos resultados deste estudo está disponível mediante solicitação ao autor correspondente [Júlia Rudzinski Roveri]. O conjunto de dados não está publicamente disponível devido ao fato de conter informação que compromete a privacidade dos participantes da pesquisa.

# Translation: La experiencia de los adolescentes/adultos jóvenes con enfermedad renal crónica y sus familias durante la transición de la atención

**DOI:** 10.1590/1980-220X-REEUSP-2024-0365es

**Authors:** Júlia Rudzinski Roveri, Maiara Rodrigues dos Santos, Ana Márcia Chiaradia Mendes-Castillo, Aline Oliveira Silveira, Regina Szylit, Maira Deguer Misko

**Affiliations:** 1 Universidade Estadual de Campinas, Faculdade de Enfermagem, Campinas, SP, Brasil.; 2 Universidade de São Paulo, Escola de Enfermagem, Departamento de Enfermagem Materno-Infantil e Psiquiátrica, São Paulo, SP, Brasil.; 3 Universidade de Brasília, Faculdade de Ciências da Saúde, Departamento de Enfermagem, Brasília, DF, Brasil.

**Keywords:** Insuficiencia Renal Crónica, Enfermedad Crónica, Transición a la Atención de Adultos, Adolescente, Adulto Joven, Enfermería Pediátrica

## Abstract

**Objetivo:**

Comprender la experiencia de los adolescentes/jóvenes adultos con enfermedad renal crónica y sus familias durante la transición de la atención pediátrica a la atención de adultos.

**Método:**

Estudio cualitativo basado en los supuestos de la Teoría Fundamentada y el marco teórico del Interaccionismo Simbólico. Se entrevistó a ocho familias de adolescentes/adultos jóvenes diagnosticados de enfermedad renal crónica, en proceso de transición asistencial, en seguimiento en un hospital público docente durante 2019 y 2020. Los datos se obtuvieron mediante observación participante, entrevistas semiestructuradas y consulta de historias clínicas.

**Resultados:**

El análisis comparativo de los datos permitió descubrir la experiencia de la familia en el proceso, representada por las categorías «tener que seguir adelante», en la que los participantes describen la necesidad de fortalecer la independencia del adolescente/joven a partir del cambio de roles dentro de la familia, y «irrumpir en el nuevo mundo», donde se debe fomentar la autonomía del adolescente/joven.

**Conclusión:**

El estudio destacó la importancia de planes individualizados para la transición, la colaboración entre la familia y el equipo de salud, y el fortalecimiento de la independencia y el autocuidado de los adolescentes y adultos jóvenes.

## INTRODUCCIÓN

La prevalencia de enfermedades crónicas en la adolescencia ha crecido considerablemente en las últimas décadas. Se estima que aproximadamente el 10% de los adolescentes de los países occidentales viven hoy en día con enfermedades crónicas^(1)^. Los continuos avances tecnológicos en el campo de la medicina han contribuido a mejorar el pronóstico, permitiendo que estos jóvenes lleguen a la edad adulta, aumentando la necesidad de realizar la transición de la atención pediátrica al sistema sanitario orientado a los adultos^(2,3)^.

El concepto más utilizado en la literatura para el término «transición de cuidados» es el de la Society for Adolescent Medicine, que lo define como «un movimiento intencionado y planificado de adolescentes y adultos jóvenes con enfermedades crónicas desde un servicio pediátrico a servicios sanitarios orientados a adultos»^(4,5)^. El adolescente/joven adulto que vive con una enfermedad crónica, además de los cambios esperados para su edad, también tendrá que enfrentarse a cambios en su asistencia sanitaria y en la forma en que ésta se presta, lo que añade complejidad a un periodo de transición ya de por sí problemático^(6,7)^.

Aunque en los últimos años se han formulado diversas recomendaciones, la transición a la atención de adultos sigue representando un reto para los adolescentes, el personal sanitario y los familiares. Este proceso requiere un cambio en la dinámica familiar, en la que los miembros de la familia asumen nuevas funciones y responsabilidades^(8)^. Muchos adolescentes viven la transición de forma desarticulada, enfrentándose a dificultades tanto para abandonar el entorno pediátrico familiar como para adaptarse al entorno adulto. Los profesionales pediátricos muestran resistencia a transferir responsabilidades a los pacientes, mientras que el personal en salud de adultos no siempre está preparado para tratar con jóvenes^(1)^.

La transición a la atención de adultos debe tener en cuenta la complejidad de la enfermedad, el contexto familiar y la disposición del adolescente a responsabilizarse de su salud, en lugar de basarse únicamente en la edad cronológica^(9)^. Una transición brusca, sin planificación ni coordinación, puede causar indefensión, poner en peligro la adherencia al tratamiento y empeorar el cuadro clínico. Por ello, es fundamental que el equipo sanitario elabore un plan gradual y estructurado que garantice la continuidad asistencial, favoreciendo una mejor adherencia al tratamiento y previniendo futuras complicaciones derivadas de la enfermedad crónica^(6,7)^.

Para que esto sea posible, se entiende que es extremadamente importante conocer los sentimientos y las expectativas del adolescente y de su familia que pronto pasarán a la atención de adultos, así como la experiencia del joven adulto que ya está siendo controlado en la consulta externa de adultos y de su familia. Esperamos que este conocimiento permita a los profesionales de la salud elaborar un programa de transición asistencial individualizado y organizado, teniendo en cuenta la dinámica familiar, la enfermedad y el entorno sociocultural en el que se inserta el adolescente^(10,11)^.

El objetivo de este estudio fue, por tanto, conocer la experiencia de las familias y/o adolescentes/jóvenes adultos con enfermedad renal crónica durante su transición de la atención pediátrica a la atención de adultos.

## MÉTODO

### Diseño del Estudio

Se trata de un estudio cualitativo basado en los supuestos del marco metodológico de la Teoría Fundamentada, desarrollado desde la perspectiva del Interaccionismo Simbólico como marco teórico. Se eligió esta metodología por su capacidad para guiar la Recolección, organización y análisis de los datos, con el objetivo de elaborar una teoría, hipótesis o concepto a partir de la información obtenida durante la investigación. Busca extraer significado de las experiencias vividas, con énfasis en las interacciones sociales. También permite la construcción de categorías interrelacionadas que contribuyen a la formación de un marco teórico, ampliando la comprensión de los temas estudiados, explorando nuevas perspectivas y clarificando las experiencias analizadas^(12)^. Las recomendaciones del Consolidated Criteria for Reporting Qualitative Research (COREQ)^(13)^ orientan la construcción de este estudio.

### Sitio del Estudio

Los datos fueron recogidos en el Ambulatorio de Nefrología Pediátrica y en el Ambulatorio de Nefrología de Adultos, ambos pertenecientes al Centro Integrado de Nefrología (CIN), unidad que forma parte de un complejo hospitalario público de nivel terciario centrado en actividades de enseñanza e investigación, localizado en el interior del estado de São Paulo. Esta unidad es exclusiva para la atención de pacientes con enfermedad renal crónica, de los cuales 30% son pediátricos y 70% adultos. La atención ambulatoria abarca todas las modalidades terapéuticas y ofrece cuidados para todas las fases de la enfermedad renal, desde el tratamiento conservador hasta el trasplante de riñón. El equipo médico determina qué adolescentes deben pasar al servicio de adultos, utilizando como criterios principales la edad de dieciocho años y la estabilidad clínica del adolescente. Los pacientes con condiciones de salud inestables y las familias con una vulnerabilidad social o emocional significativa pueden ver pospuesta la transición de la atención hasta la estabilidad clínica o familiar, lo que puede ocurrir después de que el paciente cumpla 20 años.

### Población

Los participantes en esta investigación fueron adolescentes (de 12 a 18 años) y adultos jóvenes (de 19 a 24 años) con enfermedad renal crónica, así como sus familias. La elección de este grupo de edad pretendía abarcar la experiencia completa de la transición asistencial, considerando por igual todas las etapas de la adolescencia. Los adolescentes que todavía están recibiendo atención pediátrica pueden compartir sus expectativas para el futuro, mientras que los adultos jóvenes que ya han sido tratados en un ambulatorio de adultos pueden expresar sus sentimientos sobre la transición que ya han experimentado. Cabe destacar que todos los participantes adolescentes y adultos jóvenes estaban recibiendo tratamiento conservador en la clínica, a excepción de los miembros de las familias 4 y 5, que ya se habían sometido a un trasplante de riñón.

Actualmente, la definición de familia varía según el marco de referencia de cada individuo, teniendo en cuenta sus valores y creencias. En otras palabras, la familia es definida por sus propios miembros, independientemente de los lazos de sangre. Sin embargo, el sistema familiar no sólo está formado por personas que tienen vínculos afectivos, sino también por individuos que cumplen una función cultural y social dentro de este sistema^(14)^, y ésta fue la definición utilizada en este estudio.

Se siguieron los siguientes criterios de inclusión: ser familiar de un adolescente o adulto joven con enfermedad renal crónica que haya vivido o vaya a vivir el proceso de transición de cuidados en el CIN; ser un adolescente o adulto joven diagnosticado de enfermedad renal crónica que haya vivido o vaya a vivir el proceso de transición de cuidados en el CIN. Los criterios de exclusión fueron: familiares, adolescentes y adultos jóvenes con deficiencias neurológicas o del habla que imposibilitaran la realización de las entrevistas; adolescentes o adultos jóvenes que estuvieran hospitalizados durante la fase de recogida de datos.

Ocho familias participaron en la investigación, con un total de 14 familiares entrevistados: seis adolescentes y un adulto joven con enfermedad renal crónica, cuatro madres, dos padres y una hermana. Cabe señalar que el joven adulto de la familia 05 no participó en la investigación debido a un retraso en el desarrollo neuropsicomotor. En el caso de la familia 06, sólo el joven adulto participó en la investigación porque ya no asiste al servicio con un cuidador. Ninguna de las familias invitadas a participar en el estudio se negó. Los participantes se describen en el Cuadro 1.

**Cuadro 1:** Caracterización de los participantes en la investigación – Campinas, SP, Brasil, 2020.

Grupo Muestral	Familia	Nombre	Edad	Parentesco	Diagnóstico	Tiempo de transición
1 – Ambulatorio de Pediatría	Familia 1	Rosana	42	Madre	Síndrome Nefrótica	Sin previsión
		Marcela	17	Paciente		
	Familia 2	Clara	45	Madre	Síndrome Nefrótica	Última consulta pediatría
		Eduardo	18	Paciente		
	Familia 3	André	43	Padre	Glomerulopatía	Última consulta pediatría
		Laís	19	Paciente		
	Familia 4	Mara	48	Madre	Cistinosis Nefropática	Sin previsión
		Vitor	20	Paciente		
2 – Ambulatorio Adulto	Familia 5	Francisco	40	Padre	Insuficiencia Renal Crónica	1 mes
	Familia 6	Antônio	18	Paciente	Síndrome Nefrótico	5 meses
	Familia 7	Paula	46	Madre	Insuficiencia Renal Crónica	5 meses
		Valéria	18	Paciente		
	Familia 8	Milena	25	Hermana	Insuficiencia Renal Crónica	1^a^consulta
		Caio	18	Paciente		

### Definición de la Muestra

La recolección de datos finalizó cuando se alcanzó la saturación teórica, etapa en la que la recurrencia y la ausencia de nuevos elementos indican que la profundización en la teoría ha sido suficiente. Este concepto se utiliza ampliamente en la investigación cualitativa en salud. Así, el número de participantes no fue predefinido, sino que se determinó a medida que se recogían y analizaban los datos^(15)^.

El grupo de muestra denominado «Consulta externa pediátrica» estaba formado por cuatro familias que estaban en seguimiento en la consulta externa de nefrología pediátrica, pero que ya habían sido informadas por el equipo de salud de que el proceso de transición a la atención de adultos estaba a punto de tener lugar. El objetivo de esta primera etapa era comprender cómo vivían el adolescente/joven adulto y su familia la transición de la atención cuando aún era una posibilidad, y cuáles eran sus sentimientos y expectativas. El segundo grupo de la muestra estaba formado por cuatro familias que ya habían vivido el proceso de transición a la atención de adultos y estaban iniciando o ya estaban siendo controladas en el ambulatorio de adultos, lo que fue fundamental para ampliar nuestra visión de la experiencia de la familia en el proceso de transición. Es importante destacar que los grupos de muestra eran distintos y no se solapaban; en otras palabras, las familias del primer grupo de muestra no participaron en la investigación en la segunda fase y viceversa.

### Recolección de Datos

La recolección de datos se realizó entre agosto de 2019 y marzo de 2020, mediante los métodos de observación participante, entrevistas semiestructuradas y consulta de historias clínicas.

La observación participante es una estrategia que permite al investigador sumergirse en la situación investigada, siguiendo expresiones verbales y no verbales, además de ayudar a identificar detalles que, hasta ese momento, no habían sido notados o reportados durante las entrevistas. La observación se llevó a cabo tanto en la sala de espera como durante las citas de enfermería que preceden a las consultas médicas. Se acompañó a cada familia durante una media de 60 minutos. Estas observaciones se registraron en un diario de campo en el momento en que se realizaron, lo que se conoce como «notas de observación».

La entrevista semiestructurada fue realizada por la investigadora responsable y constó de dos secciones: la primera destinada a obtener información sociodemográfica y caracterizar a la familia, y la segunda consistente en la pregunta guía «¿Cómo ha sido para usted vivir con su hijo/familia el proceso de transición de la atención de un servicio pediátrico a un servicio de adultos?». Los discursos surgidos de esta pregunta se impregnaron de nuevas cuestiones, como «¿Cómo le hizo sentir esta situación?» y «¿Qué cree que podría haberle ayudado en esta situación/momento?», con el fin de profundizar en la descripción de la experiencia. Las entrevistas duraron una media de 30 a 40 minutos y se realizaron una sola vez con cada participante.

El contacto con los participantes tuvo lugar en la sala de espera del ambulatorio pediátrico o de adultos, mientras esperaban la atención médica o de enfermería. El investigador fue presentado a la familia por los profesionales del ambulatorio, y no estableció ningún vínculo o contacto previo con estas familias, ni antes ni después de la entrevista. Sólo la investigadora recogió los datos, y no tenía ningún vínculo con el lugar del estudio, lo que contribuyó a minimizar posibles incomodidades o sesgos en el acceso a los sujetos de la investigación.

La entrevista con cada familia se realizó individualmente, respetando la intimidad del entrevistado, en el lugar y en el momento de preferencia de la familia. Todos los entrevistados optaron por ser entrevistados en la clínica donde eran atendidos, en una sala privada previamente reservada por la investigadora para este fin. Se dio a la familia la opción de realizar las entrevistas sin la presencia del adolescente/joven adulto y también se hizo a la inversa, pero ninguna familia utilizó esta opción.

Se invitó a los adolescentes a participar en la entrevista, pudiendo optar por asistir con sus familiares o individualmente. Todos decidieron participar junto con sus familias y se expresaron abiertamente, compartiendo sus sentimientos y expectativas. Los participantes recibieron seudónimos para garantizar la confidencialidad de su identidad.

La investigadora principal llevó a cabo la recogida de datos en su totalidad y, para ello, recibió formación sobre la realización de entrevistas con familias, ofrecida por el grupo de investigación al que pertenece. Las entrevistas se grabaron en un dispositivo digital y se transcribieron íntegramente.

Las transcripciones fueron revisadas por miembros del grupo de investigación y los participantes no tuvieron acceso a las mismas.

También se consultaron las historias clínicas físicas y electrónicas de las pacientes para obtener información como el diagnóstico, el tiempo de seguimiento en el servicio, el tratamiento y el pronóstico de la enfermedad.

### Análisis y Tratamiento fe Los Datos

Las declaraciones grabadas se transcribieron íntegramente de forma manual y se analizaron mediante el método comparativo continuo, por el que se compararon las categorías para identificar conceptos similares o diferentes, de forma simultánea a la Recolección de datos. Las categorías fueron revisadas por los miembros del equipo.

El análisis de los datos se basó en los principios de la teoría fundamentada, mediante codificación abierta, axial y selectiva. Así, la primera etapa del proceso analítico fue la codificación abierta, que consiste en la lectura atenta por parte del investigador de cada frase pronunciada por el entrevistado, seguida de reflexión y conceptualización de la frase. En esta fase, se atribuyeron expresiones a los fragmentos de la entrevista para formar códigos. Este proceso consistió en analizar la entrevista frase por frase y palabra por palabra, teniendo en cuenta las preguntas y los objetivos de la investigación^(16)^. A medida que se respondía a las preguntas, fue posible identificar las primeras propiedades, que permitieron formar las categorías presentadas en este trabajo.

La codificación axial fue la segunda fase del análisis, caracterizada por el proceso de vinculación de las categorías con sus subcategorías, con el objetivo de generar explicaciones más detalladas y completas de los fenómenos. Dado que las conexiones entre categorías pueden ser bastante implícitas, Strauss y Corbin^(16)^ sugieren elaborar un esquema que pueda utilizarse para estructurar las relaciones emergentes. En este estudio, se adoptó el denominado tipo «proceso» para la codificación axial. Strauss y Corbin^(16)^ lo definen como una serie de secuencias evolutivas de acción/interacción que tienen lugar a lo largo del tiempo y el espacio, cambiando o permaneciendo constantes en determinados momentos en respuesta a la situación o el contexto. Así, el proceso en los datos está representado por acontecimientos y sucesos que pueden o no desarrollarse en secuencias continuas.

La codificación selectiva es la tercera fase del análisis, en la que intentamos integrar y refinar la teoría. Al igual que en las fases anteriores del análisis, esta integración implica una interacción entre el analista y los datos, ya que abarca la evolución del pensamiento a lo largo del tiempo, mediante la inmersión en los datos y el conjunto de resultados obtenidos.

A continuación, se compararon los datos en busca de similitudes y diferencias. Este proceso permitió analizar detalladamente cómo se utilizaban las palabras y frases, asociándolas a sentimientos y experiencias, lo que ayudó a interpretar los datos, formando agrupaciones de conceptos más abstractos, que se denominan categorías y se ejemplifican en el Cuadro 2.

**Cuadro 2:** Ejemplo de categorización – Campinas, SP, Brasil, 2020.

Códigos	Categoría
– Quedar a la expectativa. – Tener un poco de ansiedad. – Saber que no son los mismos profesionales, pero creer que tendrán el mismo ánimo. – Creer que aportará más. – Recibir la noticia de que iba al ambulatorio de adultos. – Expectativa con la transición.	TENER QUE PASAR A LA SIGUIENTE FASE

### Aspectos Éticos

El proyecto fue sometido al Comité de Ética en Investigación de la Universidad Estadual de Campinas y aprobado con el número de dictamen 3.276.133. Cabe destacar que los aspectos de la Resolución 466/2012 del Consejo Nacional de Salud fueron estrictamente respetados. Todos los participantes fueron informados sobre los objetivos del estudio, los procedimientos y métodos, especialmente en relación con el uso de una grabadora durante las entrevistas, la garantía de confidencialidad de la información, el derecho al anonimato y la libertad de elegir si participar o no en la investigación. Los adolescentes y sus familiares fueron invitados a participar en el estudio y, tras la lectura del Formulario de Consentimiento Libre e Informado (para los participantes menores de 18 años) y del Formulario de Consentimiento Libre e Informado (para los mayores de 18 años y todos los familiares), se aclararon dudas y se solicitaron las firmas de los implicados.

## RESULTADOS

Los significados dados por los familiares a la experiencia de transición para el cuidado de adultos fueron comprendidos después de evaluar y comparar continuamente los datos. Así, el proceso que refleja la experiencia de la familia se compone de dos categorías: «Tener que pasar a la siguiente fase» y «Explorar un nuevo mundo».

### Tener Que Pasar a la Siguiente Fase

La experiencia de la familia de adolescentes y/o adultos jóvenes con enfermedad renal crónica durante el proceso de transición asistencial está estrechamente vinculada al proceso de adaptación familiar, especialmente cuando el diagnóstico se produce en la infancia. Este proceso de adaptación implica una combinación de factores emocionales y prácticos, que reflejan tanto los cambios en la vida cotidiana de la familia como la gestión de las emociones ante una enfermedad crónica y potencialmente debilitante.

Muchas familias informaron de una reducción de su fragilidad emocional cuando empezaron a ser controladas por el servicio de pediatría. Este apoyo del equipo sanitario les ayudó a hacer frente a la enfermedad y a establecer una rutina de tratamiento. La socialización en el ambulatorio ha reforzado los lazos afectivos entre los familiares y el personal sanitario, reduciendo la carga emocional que conlleva la enfermedad.

*“Aquí está el lado personal. Porque llevo viniendo aquí 16 años, así que me conocen desde que era pequeño». [Eduardo, 18 años].*

*“Todas las enfermeras son amigas suyas, todas le conocen desde que era pequeño: ‘¡Eh, Joãozinho, ya has crecido, ahora vas a ser adulto! ‘. Y él: ‘Vaya, ¿vas a pasar el examen de adulto? No, ¡no puedes!’» [Milena, hermana]*

*“Marcela espera en la sala de espera con su madre, mirando el móvil. La llama la enfermera y entra sola en la consulta. Dentro de la consulta, Marcela ya conoce el procedimiento: se quita los zapatos y se ata la blusa a la cintura. Se sube a la báscula y la enfermera, mientras anota su peso, le pregunta por su novio, con el que Marcela ha iniciado recientemente una relación. Marcela empieza a hablarle de su último fin de semana y la conversación fluye como la de dos amigas» [notas de campo tomadas por la investigadora a partir de la observación participante].*

Durante la transición de los cuidados, la familia romperá los vínculos afectivos. Al dejar la pediatría, la familia dejará atrás años de interacciones sociales construidas, experiencias y aprendizajes compartidos, momentos tristes y felices que conformaron sus trayectorias, para entrar en un mundo con nuevos significados. Las familias manifiestan inseguridad por la falta de apoyo e información, así como miedo y tristeza ante la mudanza, sobre todo cuando no disponen de las instrucciones y normas necesarias para afrontar este cambio.

*“No quiero ir* (a la clínica de adultos)*. Me siento triste, no quiero ir. Ni siquiera lo pienso, no quiero ir allí, me gusta estar aquí». [Marcela, 17 años].*

*“Sólo el miedo. No sé cómo es allá, no sé cómo va a ser. Sólo miedo». [Laís, 19 años].*

*“El equipo médico que está al lado de la sala infantil es muy majo, hay un médico allí, vaya, muy bueno. Si pudiera, los dejaría aquí (en la clínica infantil), se ve que son más cariñosos. No me gustaban mucho en la clínica de adultos, donde no me hacían mucho caso». [Francisco, padre]*

La posibilidad de cambios en el tratamiento y en las rutinas de salud hace que la familia sienta que el proceso de transición asistencial puede poner en peligro la continuidad del tratamiento, o añadir nuevas restricciones a la vida diaria, como es el caso de la hemodiálisis.

*“Habla mucho de ello: “¿Va a haber mucha diferencia?” (...) ¿Voy a cambiar alguna medicación? ¿Voy a cambiar mi rutina?’ Aún no lo sabe» [Milena, hermana]. [Milena, hermana] «Creo que mi mayor miedo es llegar allí y, ya sabes, la hemodiálisis. Porque aquí, no sé cómo es allí, pero aquí dan medicinas, porque los cuidados que se dan a los niños son diferentes a los que se dan a los adultos. Intentan dar intimidad al niño para que siga creciendo, pero allí no». [Laís, 19 años].*

No sólo los procedimientos y las rutinas son diferentes en la clínica de adultos, sino también el espacio físico y las interacciones personales en el nuevo entorno, lo que tiene un impacto significativo en la familia. Esto se debe a que las relaciones tienden a ser más distantes y las citas más protocolizadas. Muchas familias afirman no estar preparadas para afrontar este cambio de equipo y de ambulatorio, a la vez que se sienten presionadas para seguir adelante y aceptar la transición.

El joven adulto y su familia tendrán que interactuar con un equipo sanitario nuevo y desconocido, con el que tendrán que establecer relaciones de confianza similares a las vividas en el entorno pediátrico. Sin embargo, las familias temen que estas nuevas relaciones se vuelvan más difíciles y rígidas. Además, la dificultad natural de interacción típica de la adolescencia, agravada por la presencia de una enfermedad crónica, es una preocupación expresada a menudo por las familias que viven este proceso.

*“Ya te conocen (los pediatras), ya saben quién eres, ya conocen tu forma de ser. A veces estás alegre, a veces estás abatido. Así que parece que allí ya te haces amigo. En el otro lado (ambulatorios de adultos) he estado varias veces, pero es todo un poco árido». [Mara, madre]*

*“No creo que el médico (del ambulatorio de adultos) te trate igual. A lo mejor es un poco más estricto, no le preguntes a su personal». [Eduardo, 18 años]*

*“Al principio me pareció un poco raro, un poco diferente. Aquí (pediatría) lo hacían de una manera, allí es de otra. La forma de pesar es diferente». Antônio, 18 años.*

Este proceso genera ansiedad y sufrimiento en las familias, que se dan cuenta en este momento de que tendrán que desarrollar nuevas habilidades para que el adolescente o joven pueda ganar voz y ser el narrador de su historia. El miedo a no establecer vínculos con el nuevo equipo está profundamente relacionado con el miedo a presentarse ante un grupo hasta entonces desconocido.

*“Es que mi madre siempre habla por mí. Ahora intento hablar conmigo misma. Es un poco extraño». [Valéria, 18 años].*

Las familias temen no poder replicar los procedimientos y tratamientos realizados durante el seguimiento nefropediátrico para el equipo de adultos, lo que podría comprometer el seguimiento y la salud del adolescente. La continuidad del tratamiento parece estar en riesgo, y sienten que tendrán que afrontar este viaje solos y con poca información.

*“En cuanto a la transición (...) yo no soy médico para contarte, para contarte mi historia sobre lo que pasó, ¿no? Sé que ocurrió, pero no puedo hablar de la parte técnica. Decir: ‘empezó con síndrome nefrótico, pasó todo esto, tiene un caso así’, contar mi caso, ¿sabes? Y tenerlo listo para que el médico lo coja y diga: ‘¡Ah, este es Eduardo! Ya sé quién es Eduardo. Así me sentiría un poco más tranquilo, porque él ya conocería un poco mi historia. Por supuesto, no lo sabría todo, porque acaba de empezar el asesoramiento». [Eduardo, 18 años].*

En la categoría «Tener que pasar a la siguiente fase», la familia se enfrenta a la inevitable transición del cuidado, intentando construir una nueva identidad mientras interactúa con personas y situaciones desconocidas. A lo largo de este proceso, la familia se da cuenta de la necesidad de apoyar la independencia del adolescente, y busca establecer nuestros símbolos a partir de las nuevas relaciones que comienzan a establecerse, garantizando así la continuidad de los cuidados. Dicho esto, ya no hay vuelta atrás y los adolescentes y/o jóvenes adultos y sus familias comienzan a vivir un nuevo momento, a partir de la transición de los cuidados, representada por la segunda categoría, denominada «Explorar un nuevo mundo».

### Explorar un Nuevo Mundo

La transición a la atención de adultos representa el inicio de una nueva era en la vida de la familia y es uno de los mayores obstáculos desde el diagnóstico. La categoría «Explorar el nuevo mundo» representa los esfuerzos de la familia por reunir las herramientas indispensables para adaptarse a un contexto diferente, caracterizado por nuevas estructuras, normas y rutinas. Este periodo también destaca por la reorganización de los roles familiares, que deben ajustarse a las exigencias de esta nueva realidad.

Con el diagnóstico del niño, la familia se reorganiza para que uno de sus miembros, normalmente la madre, asuma el papel central del cuidado y se dedique casi en exclusiva al niño, supervisando el tratamiento y el seguimiento sanitario. En este proceso, estas madres pasan a ocupar un nuevo papel en la dinámica familiar: el de cuidadoras de un niño con una enfermedad crónica. Esta configuración suele durar toda la infancia, pero en la transición a la edad adulta, muchas madres reanudan sus actividades profesionales y tienen que transferir gradualmente el control y la responsabilidad del tratamiento a sus hijos, ahora adolescentes, retándoles a responsabilizarse de su propia condición médica.

*«Hace dos meses que volví a mi rutina normal, porque hasta entonces... antes de que se enfermara, trabajaba, después no... hacía algo para llenar el tiempo (...) pero ahora estoy trabajando de verdad» [Clara, madre]. [Clara, madre]*

*“(Mamá) lo controla todo, se asegura de que los medicamentos se cortan en los miligramos adecuados, se mantiene al tanto del horario (...) No sé si es la fase adolescente, pero él es muy reacio. Así que tienes que estar encima de él».[Milena, hermana]*

Al entrar en la edad adulta, los adolescentes adquieren responsabilidades y autonomía, aunque éstas no siempre van acompañadas de la madurez emocional o la disposición necesarias para tomar las riendas de la situación. La transición de los cuidados les obliga a responsabilizarse de su propia enfermedad y tratamiento, así como de las consecuencias de no seguir el tratamiento. En esta fase, la familia suele cuestionar la disposición del adolescente a asumir un papel más activo en su cuidado, al tiempo que teme un posible empeoramiento del cuadro clínico. Muchos miembros de la familia se sienten inseguros e incluso amenazados por la posibilidad de perder el control sobre la salud del adolescente, lo que puede dar lugar a preocupaciones sobre la continuidad del tratamiento y el riesgo de empeoramiento del cuadro clínico.

*“Es extraño que la dejara venir sola. Porque el día que me dijo: ‘¡Mamá, voy a ir al adulto y tú no puedes ir!’ Pero espera, ¿cuánto tiempo he estado con ella? La he cuidado, la he medicado, etc. ‘Pero mamá, tengo 18 años, ya es hora de cortar el cordón, ¿no?» [Paula, madre].*

*«Hoy se va a sentar en una habitación con un médico, la primera vez que voy con él, pero puede ser que la próxima vez no entre, así que tendré que creer que todo lo que le diga el médico me lo tendrá que transmitir a mí o a mi mamá» [Milena, hermana]. [Milena, hermana].*

*“Normalmente mi madre habla por mí. Ahora intento hablar sola. Es un poco extraño». [Valéria, 18 años].*

En general, los familiares describen el ambulatorio de adultos como «la vida real», mientras que la pediatría se presenta como un mundo «imaginario». Estos familiares traen consigo la angustia de ver que sus pequeños han crecido y entran en el mundo real y tienen que asumir el control de su enfermedad.

*“Es una nueva fase de la vida para ella, que creo que es más difícil. Su adaptación, su adaptación allí. Porque va a tener que tratar con la gente, todo ese rollo infantil, preadolescente, se acabó. Lo de adulto es más serio, en todos los sentidos, verdad, no sólo aquí» [Rosana, mamá]. [Rosana, mamá].*

En este cambio de roles, los familiares ceden el protagonismo en el cuidado y asumen el papel de apoyo, mientras que el adolescente se vuelve hacia sí mismo en busca de herramientas y recursos para desarrollar su autonomía y afrontar el miedo a no sentirse preparado para este momento. En el entorno adulto, los familiares se verán como un apoyo, pero será el joven adulto el que tendrá que responder de sus decisiones y comportamiento ante la enfermedad. Mencionan su temor a la presión que imaginan que recibirán cuando se produzca realmente la transición de la atención pediátrica a la de adultos.

*“Aquí (pediatría) era como: ‘Vamos, todavía no vas a tener el trabajo. El peso no recae en ti, sino en tu cuidador’. Pero ahora el peso va para mí. Algo será culpa mía». [Caio, 18 años]*

*“A partir del momento en que cumpla 18 años, cambiarán muchas cosas en su vida. Tendrá que enfrentarse a las cosas de frente, ya no es una niña, ya no tiene cierta ventaja. Las cosas son más serias». [Rosana, mamá]*

*“Ahora la responsabilidad es suya. Cuando le acompañamos, cuando estamos cerca, es más fácil, porque acude a nosotros, le exigimos y estamos pendientes. Ahora, cuando es para él, para que él tome (...) Lo siento por él, que ahora ve que es su responsabilidad. No es cada vez que vamos a poder entrar, que vamos a poder acompañarlo, que ahora es su responsabilidad. Así que un poco será bueno para su madurez». [Milena, hermana].*

Este proceso analítico explora, en la experiencia de la familia, la dinámica entre afrontar la inevitable transición al ambulatorio de adultos y lidiar con la ruptura de los lazos establecidos en el servicio pediátrico. Esta ruptura se acompaña del miedo a las posibles consecuencias, como la interrupción del tratamiento y el empeoramiento del cuadro clínico del adolescente. En este contexto, el adolescente o joven adulto surge como protagonista de su tratamiento, buscando mayor autonomía e independencia.

## DISCUSIÓN

Los resultados obtenidos en este estudio amplían nuestra comprensión de cómo los adolescentes y adultos jóvenes con enfermedad renal crónica, junto con sus familias, experimentan la transición de la atención pediátrica a la atención de adultos. La experiencia familiar descrita trasciende un simple cambio de entorno, convirtiéndose en un proceso complejo, permeado por cuestiones relacionadas con el desarrollo del adolescente, el cambio de roles dentro de la dinámica familiar, la continuidad de la atención sanitaria, el pronóstico de la enfermedad, entre otras.

Los familiares y adolescentes entrevistados en este estudio describieron la transición de cuidados como un proceso inevitable y muchas veces no deseado, convirtiéndose en un desafío que la familia debe superar sin haber sido preparada para ello de antemano. Sentimientos como la tristeza por la ruptura de los lazos afectivos con los profesionales pediátricos y la ansiedad derivada de la dificultad para establecer relaciones de confianza con el equipo asistencial adulto fueron relatados por los participantes en la encuesta. Este hallazgo está respaldado por otros estudios^(8,17,18)^ que muestran que las diferencias culturales entre la atención pediátrica y la de adultos crean barreras significativas para la continuidad de la atención durante el proceso de transición.

Tanto los adolescentes como los familiares destacaron la importancia del apoyo emocional y clínico por parte del equipo sanitario, señalando que una buena relación con el equipo favorece la aceptación del diagnóstico, la adherencia al tratamiento y un mejor pronóstico de salud. Una de las principales cuestiones planteadas en este estudio fue el temor a no poder establecer relaciones de confianza con el equipo de adultos, lo que podría suponer una pérdida de atención sanitaria y un empeoramiento del cuadro clínico. La importancia del vínculo con los equipos asistenciales ya ha sido destacada en varios estudios internacionales^(1,17–19)^, lo que refuerza la necesidad de que los equipos asistenciales analicen detenidamente esta cuestión a la hora de elaborar los planes de transición asistencial.

Según Rosen^(20)^, la atención pediátrica es diferente de la atención a adultos por una razón funcional: los cuidadores desempeñan un papel central en el tratamiento, y el trabajo del equipo debe ser de apoyo. Esta particularidad, sin embargo, se vuelve disfuncional cuando se trata de adolescentes mayores y adultos jóvenes, ya que deben responsabilizarse de su propio cuidado y debe fomentarse el desarrollo de la autonomía. En este contexto, los papeles de los cuidadores y de los adolescentes deben redefinirse dentro de la dinámica familiar^(20)^.

Este estudio reveló que la familia desea participar activamente en la fase de transición de los cuidados, reconociendo la importancia de transferir gradualmente la responsabilidad del tratamiento y de ayudar al adolescente a alcanzar la autonomía y la independencia. Esta transferencia del cuidado parental al autocuidado del joven adulto es un acontecimiento crítico para toda la familia y fue mencionado tanto por los participantes en este estudio como en investigaciones anteriores sobre el tema^(1,8,17,21)^.

Aunque reconocen la necesidad de transferir los cuidados dentro de la dinámica familiar, muchos miembros de la familia muestran preocupación y miedo a permitir que el joven tome el control de su tratamiento. Esta reticencia se deriva de la falta de confianza en la capacidad del joven para responsabilizarse plenamente de su salud, un aspecto que también se discute en la literatura^(17)^. Esta expectativa por parte de los familiares hacia este adolescente genera ansiedad en una persona que está construyendo una identidad, que tiene dificultades para confiar en sus propias habilidades y en su capacidad para establecer lazos de confianza con el equipo de salud del ambulatorio de adultos. En el momento de la transición asistencial, la familia quiere confiar en que el adolescente será capaz de asumir por sí solo toda la responsabilidad de su tratamiento, y ser independiente y autosuficiente en sus decisiones^(17)^.

Este estudio reveló que a muchos adolescentes les preocupa no conocer suficientemente bien su estado clínico y no ser capaces de transmitir la información necesaria al médico del equipo de adultos, ya que ahora acudirán solos a las citas médicas. El miedo a no adaptarse a este cambio de equipo y a la forma en que se llevará a cabo su tratamiento provoca ansiedad en estos jóvenes, un sentimiento reportado por los participantes en otros estudios similares^(1,8,17,21)^.

Algunos factores que facilitarían el proceso de transición asistencial fueron señalados por las familias en este estudio y corroborados por hallazgos en la literatura, como tender puentes entre servicios, mejorar la comunicación entre el equipo pediátrico y el de adultos^(17)^, visitar el servicio de adultos y conocer al nuevo equipo sanitario antes de la transición, participar en la elaboración de un proyecto de transición bien estructurado junto con los profesionales sanitarios, hablar previamente con el equipo sobre las diferencias entre los servicios y tener la oportunidad de establecer vínculos tempranos con el equipo sanitario de adultos mientras aún están en seguimiento pediátrico^(22)^.

Así, para esta investigación, basándonos en el marco teórico del Interaccionismo Simbólico, consideramos que los significados para el proceso de transición asistencial se construyen a partir de las interacciones sociales de la familia, ya sean estas interacciones entre los miembros de la familia, con el equipo sanitario o con otros pacientes. Tener un hijo en el proceso de transición asistencial hace que la familia desarrolle significados y perspectivas que surgen de la interacción social. Estudiar a esta familia nos permite comprender los significados que atribuyen a estos símbolos y sus consiguientes comportamientos.

## CONCLUSIÓN

Los hallazgos de este estudio corroboran que la transición del cuidado es una fase desafiante para los adolescentes/jóvenes adultos y sus familias, ya que implica necesariamente un cambio de roles dentro de la familia. Es el momento en que los cuidadores ceden el protagonismo, cediendo gradualmente el control de la situación al adolescente y asumiendo la posición de apoyo. El adolescente, a su vez, debe responsabilizarse de su tratamiento y atención sanitaria, participando activamente en las decisiones que afectan a su salud. La implicación de la familia y del equipo interdisciplinar es fundamental en este proceso y en el éxito de la transición asistencial, manteniendo la adherencia y la calidad del tratamiento.

La importancia innegable de este tema no puede ser exagerada, dado el escenario epidemiológico actual, donde las enfermedades crónico-degenerativas son cada vez más prominentes y estos niños tienen más probabilidades de llegar a la adolescencia y a la edad adulta, y son beneficiarios directos de planes estructurados y equipos preparados para liderar la transición de la pediatría a los servicios de adultos, teniendo en cuenta las particularidades que impregnan la fase adolescente y su transición a la edad adulta.

Esta información resalta la importancia de crear políticas públicas de salud con iniciativas dirigidas a la transición de cuidados, que deben ser planificadas por los servicios teniendo en cuenta la composición de los equipos de transición de cuidados y la elaboración de un plan individualizado para el adolescente y su familia, involucrándolos en su construcción y toma de decisiones, proporcionando información compartida, reduciendo miedos y ansiedad, así como actuando para estimular la autonomía de este adolescente y/o joven adulto en la gestión de su tratamiento.

Entre las restricciones del estudio está el hecho de que la investigación fue realizada en un único servicio público de salud, por lo que no fue posible comparar las experiencias de pacientes tratados en diferentes lugares, o en instituciones de salud privadas. Entrevistar a pacientes de diferentes entornos socioculturales y diagnósticos, así como a adultos jóvenes que habían hecho la transición más recientemente, también fueron factores limitantes. Se recomienda que en el futuro se lleven a cabo investigaciones que analicen diferentes contextos socioculturales para ampliar los conocimientos sobre el tema.

## Data Availability

Todo el conjunto de datos que respalda los resultados de este estudio está disponible a solicitud del autor correspondiente [Júlia Rudzinski Roveri]. El conjunto de datos no está disponible públicamente debido a que contiene información que compromete la privacidad de los participantes de la investigación.
